# Instantaneous synthesis and full characterization of organic–inorganic laccase-cobalt phosphate hybrid nanoflowers

**DOI:** 10.1038/s41598-022-13490-w

**Published:** 2022-06-03

**Authors:** Khashayar Vojdanitalab, Hossein Jafari-Nodoushan, Somayeh Mojtabavi, Mahtab Shokri, Hoda Jahandar, Mohammad Ali Faramarzi

**Affiliations:** 1 Department of Pharmaceutical Biotechnology, Faculty of Pharmacy & Biotechnology Research Center, Tehran University of Medical Sciences, P.O. Box 14155−6451, Tehran, 1417614411 Iran; 2 Pharmaceutical Sciences Research Center, Tehran Medical Sciences Branch, Islamic Azad University, Tehran, Iran

**Keywords:** Biochemistry, Biotechnology, Environmental sciences, Materials science

## Abstract

A novel approach termed the "concentrated method" was developed for the instant fabrication of laccase@Co_3_(PO_4_)_2_•hybrid nanoflowers (HNFs). The constructed HNFs were obtained by optimizing the concentration of cobalt chloride and phosphate buffer to reach the highest activity recovery. The incorporation of 30 mM CoCl_2_ and 160 mM phosphate buffer (pH 7.4) resulted in a fast anisotropic growth of the nanomaterials. The purposed method did not involve harsh conditions and prolonged incubation of precursors, as the most reported approaches for the synthesis of HNFs. The catalytic efficiency of the immobilized and free laccase was 460 and 400 M^−1^S^−1^, respectively. Also, the enzymatic activity of the prepared biocatalyst was 113% of the free enzyme (0.5 U mL^−1^). The stability of the synthesized HNFs was enhanced by 400% at pH 6.5–9.5 and the elevated temperatures. The activity of laccase@Co_3_(PO_4_)_2_•HNFs declined to 50% of the initial value after 10 reusability cycles, indicating successful immobilization of the enzyme. Structural studies revealed a 32% increase in the α-helix content after hybridization with cobalt phosphate, which improved the activity and stability of the immobilized laccase. Furthermore, the fabricated HNFs exhibited a considerable ability to remove moxifloxacin as an emerging pollutant. The antibiotic (10 mg L^−1^) was removed by 24% and 75% after 24 h through adsorption and biodegradation, respectively. This study introduces a new method for synthesizing HNFs, which could be used for the fabrication of efficient biocatalysts, biosensors, and adsorbents for industrial, biomedical, and environmental applications.

## Introduction

The presence of micropollutants such as pharmaceuticals, personal care products (PCPs), phenolic, and estrogenic compounds in wastewaters is a growing concern^1,2^. Over the past two decades, various heterogeneous biocatalysts have been developed for environmental and industrial applications^3^. A wide range of enzymes such as laccase, manganese peroxidase, and horseradish peroxidase has been utilized for the removal of micropollutants. Although the activity of enzymes upon immobilization may decrease, the immobilized enzymes are reusable and more stable against operational conditions^4^. In this regard, enzyme immobilization technologies were developed on various organic and inorganic supports such as electrospun fibers, magnetic nanoparticles, membranes, natural polymers, etc.^5–7^.

Moxifloxacin, as a fourth-generation fluoroquinolone (FQ), is responsible for more than 34.6% of the total FQs consumption in China^8^. It is mostly used for the treatment of pneumonia and skin infections. Recently, due to its usage in severe acute respiratory syndrome coronavirus 2, moxifloxacin consumption has temporarily increased. Therefore, consumption, release, and accumulation of moxifloxacin in the environment may be threatening^8^. Moxifloxacin is also the most toxic FQs against the growth of *Pseudokirchneriella subcaptitata*^9^*.* Likewise, it exhibited significant negative effects on the growth and reproduction of *Ceriodaphnia dubia* and *Daphnia manga*^10^. Therefore, increased consumption and release of moxifloxacin in the environment may be threatening to the ecosystem and human health. The efficiency of current approaches for the elimination of antibiotics from wastewaters is limited^11^. In this regard, various techniques for the removal of antibiotics have been so far established such as biocatalysis^12^, photocatalysis^13^, electrocatalysis^14^, etc. Laccases, an oxidoreductase enzyme, has been broadly used for environmental and industrial applications^15,16^. Free and immobilized laccases have been incorporated for bioremoval of a wide range of pollutants such as bisphenol A, crystal violet, acid orange-7, levofloxacin, etc.^17–20^. However, the removal of moxifloxacin by enzymes (free or immobilized) was not reported previously.

The preparation of new platforms based on organic–inorganic hybrid materials for immobilization of enzymes is of great interest. Organic–inorganic hybrid nanoflowers (HNFs) are flower-shaped hierarchical nanostructures that were initially discovered in 2012^21^. HNFs, composed of enzyme(s) and inorganic component(s), have a large surface area, facile synthesis procedure, and hierarchical porous structures^22^. It was reported that the coordination between nitrogen atom of the organic component and the metal ion of the inorganic part is responsible for the formation of HNFs^21^. Due to their interesting properties, HNFs have been widely utilized in biocatalysis, biosensing, and biomedicine^23^. Biosensors based on HNFs provide a lower limit of detection (LOD) and faster response times due to adsorbing and concentrating contaminants surrounding enzymes^24^. HNFs-based biocatalysts have been incorporated for the removal of organic contaminations such as phenolic compounds and industrial dyes^23^. Also, due to the pH-responsive release of the immobilized biomolecule, HNFs have been incorporated as a drug delivery platform^25^. It has been reported that after incorporating enzymes into HNFs, their activity, stability, and reusability have notably increased, owing to low mass transfer limitations arising from their high surface-to-volume ratio and cooperative effects between enzymes and metal ions^26^. For instance, the incorporation of lipase into Zn_3_(PO_4_)_2_•HNFs resulted in a 147% enhancement of the enzyme activity. Also, the storage stability of the lipase-containing HNFs was three times higher than the free lipase^27^. Carbonic anhydrase (CA) was successfully immobilized into Ca_8_H_2_(PO_4_)_6_•HNFs and exhibited remarkable stability against elevated temperatures (30–80 °C). CA@Ca_8_H_2_(PO_4_)_6_•HNFs retained 100% of their initial CO_2_ hydration activity after 5 reusability cycles^28^. The addition of copper sulfate to phosphate buffer saline (PBS) in the presence of bovine serum albumin (BSA) resulted in the synthesis of the flower-shaped BSA@Cu_3_(PO_4_)_2_•HNFs^21^. Three approaches have been reported for the preparation of HNFs, including one-step precipitation^21^, ultrafast sonication^29^, and shear stress-mediated synthesis^30^. In the one-step precipitation method, a facile and straightforward strategy, fabrication of HNFs requires a long incubation time (1 ~ 3 days). As long synthesis procedure may potentially reduce the stability of the organic constituent, serious attempts have been established to shorten the assembling time. In this method, the synthesis mixture that contained Cu^2+^, phosphate ion, and BSA was sonicated for 5 min in a sonicator bath (40 kHz, 70 W), which resulted in the formation of bloomed BSA@Cu_3_(PO_4_)_2_•HNFs^29^. Introducing shear stress to the organic component and inorganic precursors to synthesize copper-based HNFs was recently reported^30,31^. The mixture of copper sulfate, BSA, and PBS was added to a vortex fluidic device (VFD) and vigorously rotated at a rotation speed of 9000 rpm with a tilt angle of 45°. After 2 min, toroidal structures with a central void appeared, which formed the final spherical HNFs morphology^31^.

Considering the fact that HNFs are at the early stages of development, the formation mechanism of HNFs has not been accurately established. However, it is believed that the formation of primary nucleation sites of metal phosphate(s) and organic molecule(s) (e.g., protein, DNA, etc.) and time-dependent anisotropic growth of these primary nucleation sites^32^ are responsible for the formation of the flower-like structures. It seems that by introducing kinetic energy to the precursors, provided by ultrasonication and vortexing, HNFs assembling time was reduced. As efficient biocatalysts, HNFs have been used for the removal of emerging contaminants such as industrial dyes. However, the ability of HNFs for the removal of antibiotics has not been reported previously^26^.

The present study aimed to develop a novel and rapid method for the synthesis of HNFs. Prolonged incubation, shear stress, and ultrasonication of the reaction mixture are the most reported approaches for the fabrication of HNFs. In this regard, the concentration of incorporated cobalt(II) ions and phosphate buffer were optimized for reaching the highest activity recovery. By the introduction of a relatively high concentration of precursors in the reaction mixture, laccase@Co_3_(PO_4_)_2_•HNFs were instantly synthesized through a rapid anisotropic growth of laccase-cobalt phosphate nanocrystals, and a new mechanism was proposed for the synthesis of HNFs. Biocatalytic properties of laccase@Co_3_(PO_4_)_2_•HNFs were investigated, and the structure of the hybridized enzyme was also characterized. The synthesized HNFs were then employed to remove moxifloxacin from aqueous media. The degradation products were identified, and their toxicity against four bacteria involved in the degradation of organic compounds was assessed.

## Experimental

### The enzyme and chemicals

Laccase from *Trametes versicolor* (0.5 U mg^−1^) and 2,2’-azino-bis(3-ethylbenzothiazoline-6-sulfonic acid) (ABTS) were purchased from Sigma-Aldrich (St. Louis, Mo, USA). Cobalt(II) chloride hexahydrate (CoCl_2_.6H_2_O) was obtained from Merck (Darmstadt, Germany). All other applied reagents and chemicals were of analytical grade without further purification. Moxifloxacin was kindly gifted from Soha Pharmaceutical Company (Tehran, Iran).

### Instant synthesis of cobalt-based hybrid nanoflowers with laccase

Regardless of the conventional methods, an instant strategy was applied for the construction of HNFs. The synthesis procedure was accomplished by the addition of 0.1 mL of CoCl_2_ solution into 0.9 mL of phosphate buffer (pH 7.4) containing 0.1 U mL^−1^ of laccase. Instantly a large number of purple precipitates appeared in the solution. Proper concentration of CoCl_2_ and molarity of phosphate buffer were subsequently optimized against highest activity recovery (n = 3, *p*-value < 0.05). The precipitates were centrifuged at 6000 g and washed three times with phosphate buffer. The purple products were marked as laccase@Co_3_(PO_4_)_2_•HNFs.

### Characterization of laccase@Co_3_(PO_4_)_2_•HNFs

Scanning electron microscopy (SEM) images (Tescan, MIRA II, Czech Republic) were applied to indicate the flower-shaped morphology of the fabricated HNFs. Energy dispersive X-Ray spectroscopy (EDX, Tescan, MIRA II, Czech Republic) and elemental mapping were incorporated to determine the composition and spatial distribution of elements in the constructed HNFs, respectively. Fourier transform infrared (FTIR) spectroscopy (Shimadzu, Equinox 55, Japan) was utilized for analyzing functional groups of organic and inorganic constituents of HNFs. The samples were dispersed in pressed KBr disks, and the spectra were recorded at 4000–400 cm^−1^. Brunauer–Emmett–Teller (BET) analysis was performed (Microtrac, BELSORP MINI X, Japan) after degassing the samples under N_2_. X-ray diffraction (XRD) analyses were carried out to identify the crystalline phase of the inorganic component using an X-ray diffractometer (Philips, PW1730, Philips, Eindhoven, Netherlands).

### Laccase activity assay

Laccase activity was spectroscopically measured followed by oxidation of ABTS as the enzyme substrate to radical cation ABTS^•+^ in phosphate buffer (10 mM, pH 4.5)^33^. Laccase@Co_3_(PO_4_)_2_•HNFs and the free enzyme were incubated with 1 mL of ABTS solution (50 µM, pH 4.5) at 40 °C in a shaker incubator. After 15 min incubation, the optical density of the supernatants was recorded at 420 nm. One unit of activity was defined as the amount of laccase capable of oxidizing 1 µmol ABTS to the colored product in 1 min. The effect of pH and temperature on the activity of both laccase@Co_3_(PO_4_)_2_•HNFs and the free form of laccase was determined in the temperature range of 25–55 °C (interval 10 °C) and pH range of 2.5–9.5 (interval 1) (n = 3, *p*-value < 0.05).

### Determination of immobilization yield and efficiency

After preparing laccase@Co_3_(PO_4_)_2_•HNFs, the concentration of residual protein in the supernatant was estimated by the bicinchoninic acid (BCA) method^34^. The amount of immobilized enzyme and the immobilization yield (IY)^7^ were calculated by Eq. (1) and Eq. (2), respectively.

1 $${\text{M}}_{{{\text{IL}}}} = {\text{M}}_{{\text{L}}} {-}{\text{C}}_{{\text{P}}} \times {\text{V}}_{{{\text{Im}}}}$$

where M_IL_ and M_L_ are the amounts of the immobilized and the added laccase (µg) to the immobilization mixture, respectively; C_P_ and V_Im_ accounted for the concentration of residual protein in the supernatant and the volume of the immobilization mixture.

2 $${\text{IY}}\left( \% \right) = \left[ {\left( {{\text{Y}}_{0} {-}{\text{Y}}_{{1}} } \right)/{\text{Y}}_{0} } \right] \times {1}00$$

where Y_0_ and Y_1_ represent the amount of the immobilized enzyme in HNFs before and after the synthesis procedure, respectively.

The immobilization efficiency (IE) was obtained by Eq. (3), where E_0_ and E_t_ demonstrate the activity of the free laccase and laccase@Co_3_(PO_4_)_2_•HNFs, respectively.

3 $${\text{IE}}\left( \% \right) = \left[ {\left( {{\text{E}}_{0} {-}{\text{E}}_{{\text{t}}} } \right)/{\text{E}}_{0} } \right] \times {1}00$$

### Evaluation of the kinetic parameters of the constructed HNFs

In order to assess the influence of immobilization on the applied enzyme, kinetic parameters of laccase@Co_3_(PO_4_)_2_•HNFs and the free laccase were evaluated. To study the effect of immobilization on the enzyme activity at different pH values, the kinetic parameters were investigated at pH 4.5 and 7.4. The activity of both the free and immobilized enzymes was determined in the presence of ABTS concentrations (20–100 µM). After the addition of ABTS solution (pH 4.5 and 7.4) to the free and immobilized enzymes, the reaction mixture was incubated for 15 min in a shaker incubator (at 40 °C), and the optical density of the supernatant was recorded at 420 nm. Lineaweaver-Burke plot was incorporated for evaluation of kinetic parameters such as Michaelis constant (*K*_m_) and maximum velocity (*V*_max_). Turnover number (*K*_cat_) and catalytic efficiency were also calculated based on Eq. (4) and Eq. (5)^35–37^; where [E] is the concentration of the enzyme.

4 $$K_{{{\text{cat}}}} = V_{{{\text{max}}}} /\left[ {\text{E}} \right]$$

5 $${\text{Catalytic}}\;{\text{efficiency}} = K_{{{\text{cat}}}} {/}K_{{\text{m}}}$$

### Reusability and stability studies

In order to evaluate the reusability of the constructed biocatalyst, repeated measurement of the activity of laccase@Co_3_(PO_4_)_2_•HNFs was examined^21^. The fabricated HNFs were incubated in 1 mL phosphate buffer (10 mM, pH 7.4) containing ABTS (0.5 mM) at 40 °C for 15 min. After centrifugation of the mixture, OD_420_ of the supernatant was recorded, and the precipitates were washed three times with phosphate buffer (10 mM, pH 7.4). The procedure was repeated to the point that the activity of HNFs dropped to below 50% of its initial value (n = 3, *p*-value < 0.05). The stability of the free laccase and HNFs was assessed as a function of recovered activity after 3 h incubation at temperatures ranging 25–55 °C (interval 10 °C) and a wide pH range of 2.5–9.5 (interval 1). Storage stability of the free laccase and HNFs were obtained by measurement of laccase activity during storage at 4 °C (n = 3, *p*-value < 0.05).

### The enzyme structural studies

In order to evaluate the conformational changes of laccase after immobilization, far-UV circular dichroism (CD) and fluorescence spectroscopies were utilized. CD spectra of laccase@Co_3_(PO_4_)_2_•HNFs and the free enzyme were recorded by a Jasco 725 spectrophotometer in a 2 mm path-length cell, and the absorbance was recorded at 190–240 nm. The fluorescence property of tryptophan is dependent on the tertiary structure of a protein. In this regard, the tryptophan fluorescence intensity of the free laccase and laccase@Co_3_(PO_4_)_2_•HNFs were recorded by a Hitachi 850 spectrofluorometer after excitation at 285 nm^33^. Finally, thermogravimetry analysis (TGA) was using performed using a thermogravimetric analyzer (Perkin Elmer STA 6000, USA) for the evaluation of thermal decomposition of both pure cobalt phosphate and laccase@Co_3_(PO_4_)_2_•HNFs. The experiment was conducted under an N_2_ atmosphere in the temperature range of 25–600 °C by a heating rate of 10 °C min^−1^.

### Bioremoval experiments

#### Bioremoval of moxifloxacin

Moxifloxacin was incubated with laccase@Co_3_(PO_4_)_2_•HNFs and 1-hydroxybenzotriazole (HBT) as the laccase mediator in phosphate buffer (10 mM, pH 4.5) at 40 °C under stirring condition (100 rpm)^38^. After determining the period of incubation, the concentration of moxifloxacin was quantified by high-performance liquid chromatography (HPLC) coupled with UV detector, and the removal percentage was calculated based on Eq. (6); as C0 and Ct represent the concentration of moxifloxacin before and after removal/adsorption experiments, respectively.

6 $${\text{Removal/adsorption}}\left( \% \right) = \left( {{\text{C}}0{-}{\text{Ct}}/{\text{C}}0} \right) \times {1}00$$

In order to assess the adsorption of moxifloxacin on the laccase@Co_3_(PO_4_)_2_•HNFs, the experiment was conducted with the inactivated HNFs, and the adsorption (%) was calculated by Eq. (5). For this, laccase@Co_3_(PO_4_)_2_•HNFs were inactivated by sequential freeze and thawing cycles. Inactivation was proved by the measurement of enzymatic activity. Finally, the degradation of moxifloxacin was calculated based on Eq. (7).

7 $${\text{Degradation}}\,\left( \% \right) = {\text{Removal}}\;\left( \% \right){-}{\text{Adsorption }}\;\left( \% \right)$$

#### Sample preparation and quantification of moxifloxacin

After the removal procedure, the pH of the reaction mixture was set to 7.95 (isoelectric point of moxifloxacin) by phosphate buffer (100 mM, pH 7.95). Then, the mixture was extracted with chloroform under vigorous vortex three times after the addition of ciprofloxacin as the internal standard. The extracted chloroform evaporated to dryness by a rotary evaporator instrument, and the remaining powder was dissolved in 1 mL of the HPLC mobile phase. A Knauer HPLC–UV system (Berlin, Germany) was incorporated for moxifloxacin quantification using a PDA 2800 detector, a Smartline 1000 pump, and ChromGate software (version 3.3.1). The mobile phase consisted of methanol (45%) and 10 mM phosphate buffer (pH 3.0) (55%). Samples were injected by Smartline autosampler 3950 to a Eurospher 100 C18 reversed-phase column (250 × 4.6 mm).

#### Identification of biotransformation products

Biotransformation products of moxifloxacin were identified by liquid chromatography coupled by mass spectroscopy (LC–MS). The apparatus was an Agilent 1200 series LC system coupled with an Agilent 6520 quadrupole time of flight tandem MS instrument featured by an electrospray ion source (Agilent, Waldbronn, Germany). A 2.1–100 mm Nucleosil 100-3 C18 HD column (Macherey–Nagel, Düren, Germany) was applied with a flow rate of 0.35 mL min^−1^. Deionized water + 0.1% formic acid (40%) and acetonitrile (60%) were used as the mobile phase.

#### Toxicity of the bioremoval procedure

Based on previous studies, elimination of toxicity is not guaranteed by the degradation of antibiotics in catalytic procedures^39^. Accordingly, the toxicity of moxifloxacin degradation products was examined against some bacteria, including *Pseudomonas aeruginosa* ATCC 9027, *Escherichia coli* ATCC 25,922, *Staphylococcus epidermidis* ATCC 49,619, and *Staphylococcus aureus* ATCC 6538, which are responsible for the bioremoval of organic pollutants in the environment. In this regard, the fresh culture of each bacterium was seeded in a nutrient broth and incubated at 37 °C overnight. Then, each bacterium was treated with a mixture of degradation products, and the optical density (OD) of each bacterium was evaluated at 600 nm after determining the incubation time periods at 37 °C. Growth inhibition of moxifloxacin degradation products, moxifloxacin as the positive control, and sterile water as the negative control were measured by plotting OD_600_ against incubation time (n = 3, *p*-value < 0.05)^20^.

#### Analysis of the experiments

The experiments were conducted in triplicate, and results were reported as mean ± standard deviation. Statistical significance among the mean value of experimental results was calculated by two-way ANOVA (± standard deviation) and *p*-value < 0.05 regarded as significant.

## Results and discussion

### Instant synthesis of laccase@Co_3_(PO_4_)_2_•HNFs

**Figure 1:**
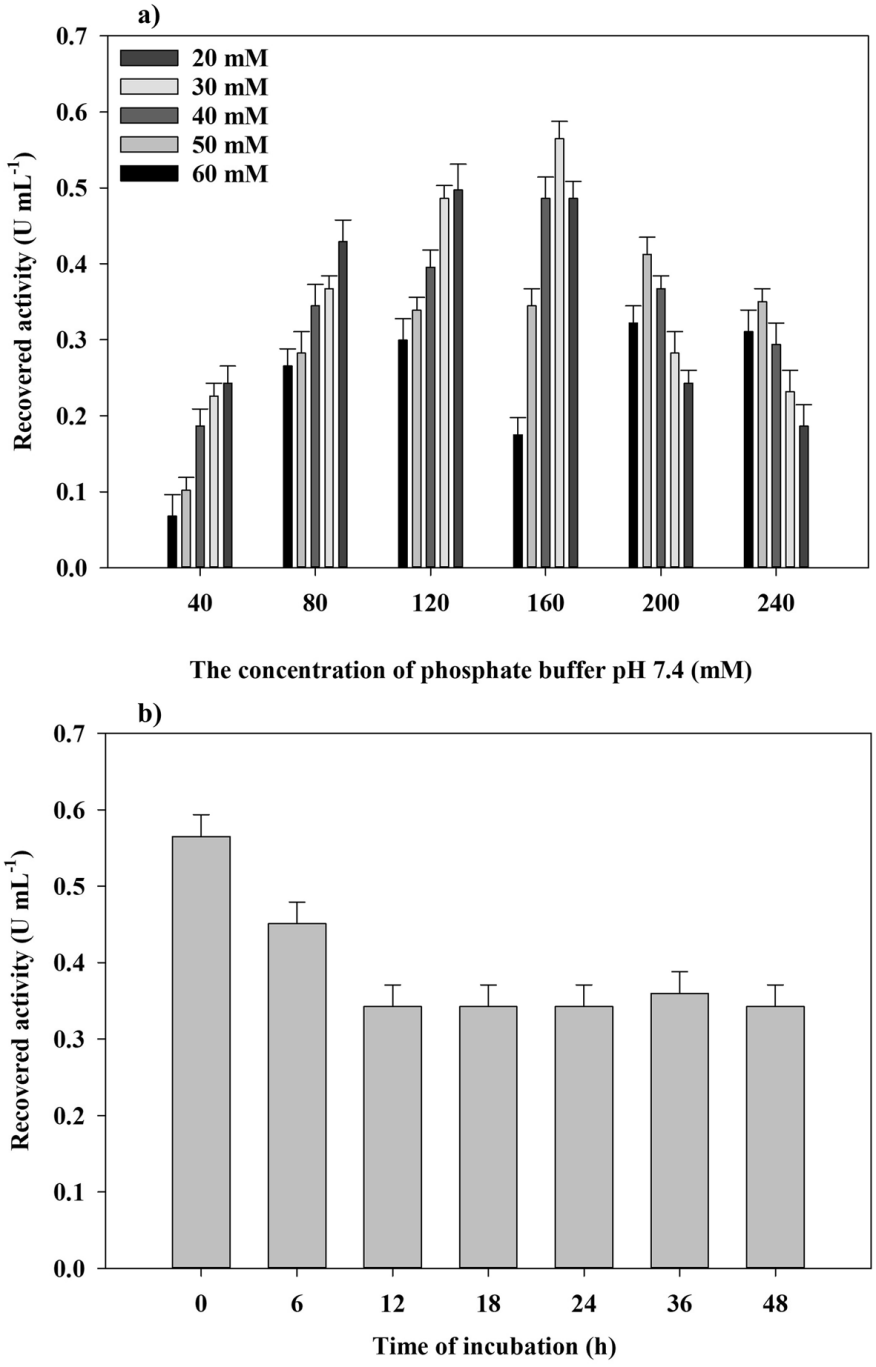
Optimization of the synthesis procedure. The synthesis of laccase@Co_3_(PO_4_)_2_•HNFs by optimizing the concentration of phosphate and cobalt (II) ions (**a**), the influence of incubation (at 25 °C) as traditional procedures for the construction of HNFs on the recovered activity of the heterogeneous biocatalyst (**b**).

HNFs have been routinely constructed with the addition of metal ions to enzyme-containing PBS followed by a 3-day incubation at room temperature, vigorous vortexing, or bath sonication, which introduces significant stress to the organic constituent^21,29,31,32^. In the present study, the concentration of CoCl_2_ and molarity of phosphate buffer (pH 7.4) were optimized to instantly fabricate HNFs and avoid incorporating stress-inducing conditions in the synthesis procedure. After the addition of CoCl_2_ to laccase-containing phosphate buffer, the purple precipitates were immediately formed. The precipitate was washed with deionized water three times and designated as laccase@Co_3_(PO_4_)_2_•HNFs. As shown in Fig. 1a, the recovered activity increased gradually at the higher molarity of phosphate buffer. However, the highest activity recovery was obtained with HNFs prepared with 0.16 M phosphate buffer and 30 mM CoCl_2_. In a study by Zheng et al.^40^, D-psicose 3-epimerase (DPEase)@Co_3_(PO_4_)_2_•HNFs were prepared by incubation of a solution containing Co^2+^ (1.9 mM), phosphate buffer (50 mM, pH 7.5), and DPEase (1 mg mL^−1^) at 4 °C for 48 h. In another study, cellobiose 2-epimerase (mutant E161D/N365P) (EDNP)@Co_3_(PO_4_)_2_•HNFs were obtained by the addition of CoCl_2_ to PBS (10 mM, pH 7.4) and incubation of the mixture for 24 h at room temperature^41^. The activity recovery was increased with the Co^2+^ concentration in the synthesis mixture, and the highest activity recovery was achieved at 2 mM Co^2+^. In the present study, the highest activity was obtained when the final concentration of Co^2+^ in the mixture was 1.36 mM. Although the concentration of incorporated Co^2+^ in the synthesis mixture was comparable to other studies, the molarity of phosphate buffer used in this study was remarkably higher. An interesting investigation on the mechanism of formation of copper phosphate nanoflowers and also BSA@Cu_3_(PO_4_)_2_•HNFs was conducted by He et al.^42^. It was reported that by increasing the concentration of the incorporated phosphate buffer (pH 7.4), the yield of copper phosphate nanoflowers increased. The same results were obtained in the present study, and by the addition of CoCl_2_ to phosphate buffer (5 mM, pH 7.4) containing laccase, negligible precipitates were formed, and the yield of laccase@Co_3_(PO_4_)_2_•HNFs was increased with the concentration of the phosphate buffer. Therefore, the yield of HNFs was increased at higher phosphate buffer concentrations. However, at phosphate buffer concentrations more than 0.16 M, the activity recovery was reduced remarkably. This could be due to the formation of a higher amount of cobalt phosphate crystals, which increases the mass transfer limitation. In order to verify the effect of incubation, the most reported method for the growth of HNFs, the optimized HNFs were incubated at 25 °C for 24–72 h. In previous studies, the incubation was replaced with bath sonication, which accelerated the growth of HNFs^29^. Therefore, the as-prepared HNFs were sonicated in a sonicator bath (60 kHz, 13.8 W) for determined time intervals. The results (Fig. 1b) demonstrated that further incubation reduced the recovered activity, which may be due to the denaturation of the enzyme. It was reported that ultrasonication increases the rate of metal phosphate self-assembly, which enhances the growth of HNFs^29^. Sonication of the reaction mixture (up to 20 min, 5 min intervals) did not increase the recovered activity. This may be due to the completion of the growth of laccase@Co_3_(PO_4_)_2_•HNFs in a relatively short period of time; therefore, further incubation or sonication did not change the recovered activity. The mentioned synthesis method is referred to as the “concentrated method” in the present study for comparison with the up to now reported and traditional procedures. The present explanation of the formation mechanism of HNFs is based on the nucleation of primary metal phosphate-protein nanocrystals and subsequent anisotropic growth of the hybrid nanoaggregates. Due to the high surface energy of the primary protein-inorganic nanocrystals, they tend to attach and form the nano-sheet structures, which finally form the ultimate structure of HNFs^22^. The growth of nucleation sites of HNFs could be facilitated by increasing the collision rate of the primary crystals^43^. It seems that the introduction of certain amounts of kinetic energy, provided by long-term incubation (24–72 h at 25 °C), bath sonication, microwave heating, and shear stress, to the synthesis mixture (organic component, metal, and phosphate ions) could induce the formation of HNFs. The number of primary nucleation sites increased by incorporating a high concentration of Cobalt(II) and phosphate ions in the reaction mixture. Hence, based on Eq. (8), higher numbers of nanocrystals possibly collide together more frequently, and HNFs were prepared very fast^43^. In Eq. (8), z is collision frequency, D is the particle diameter, ῡ represents the mean velocity of dispersed particles, N is the total number of particles, and V is the total volume of the reaction mixture.

8 $${\text{z}} = (\surd {2}\pi {\text{D}}^{{2}} \overline{\upsilon }{\text{N}}) \times {\text{V}}^{{ - {1}}}$$

Hybrid nanoflowers have been synthesized in a short period of time through vigorous vortexing^30^ and also bath sonication^29^ of the precursors, which increased the mean velocity of the dispersed particles (ῡ). Based on Eq. (8), after the increase in the mean velocity of dispersed organic–inorganic particles by vortexing or bath sonication, the collision frequency increases, which results in fast anisotropic growth of the HNFs.

### Characterization of laccase@Co_3_(PO_4_)_2_•HNFs

**Figure 2:**
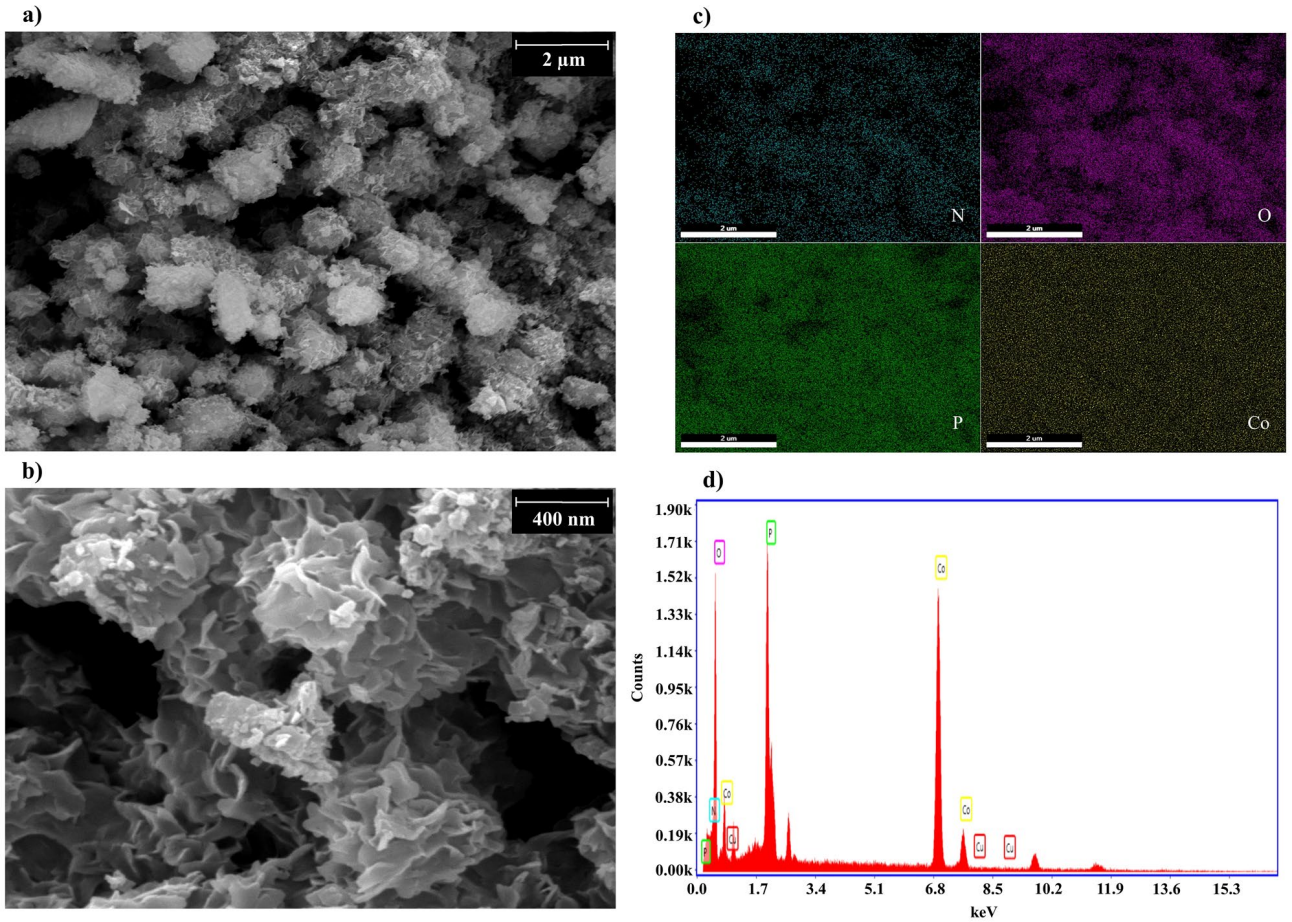
Scanning electron microscopy (SEM) imaging. SEM image (**a**,**b**), elemental map (**c**), and Energy dispersive X-Ray spectroscopy (EDX) analysis (**d**) of the constructed laccase@Co_3_(PO_4_)_2_•HNFs.

**Figure 3:**
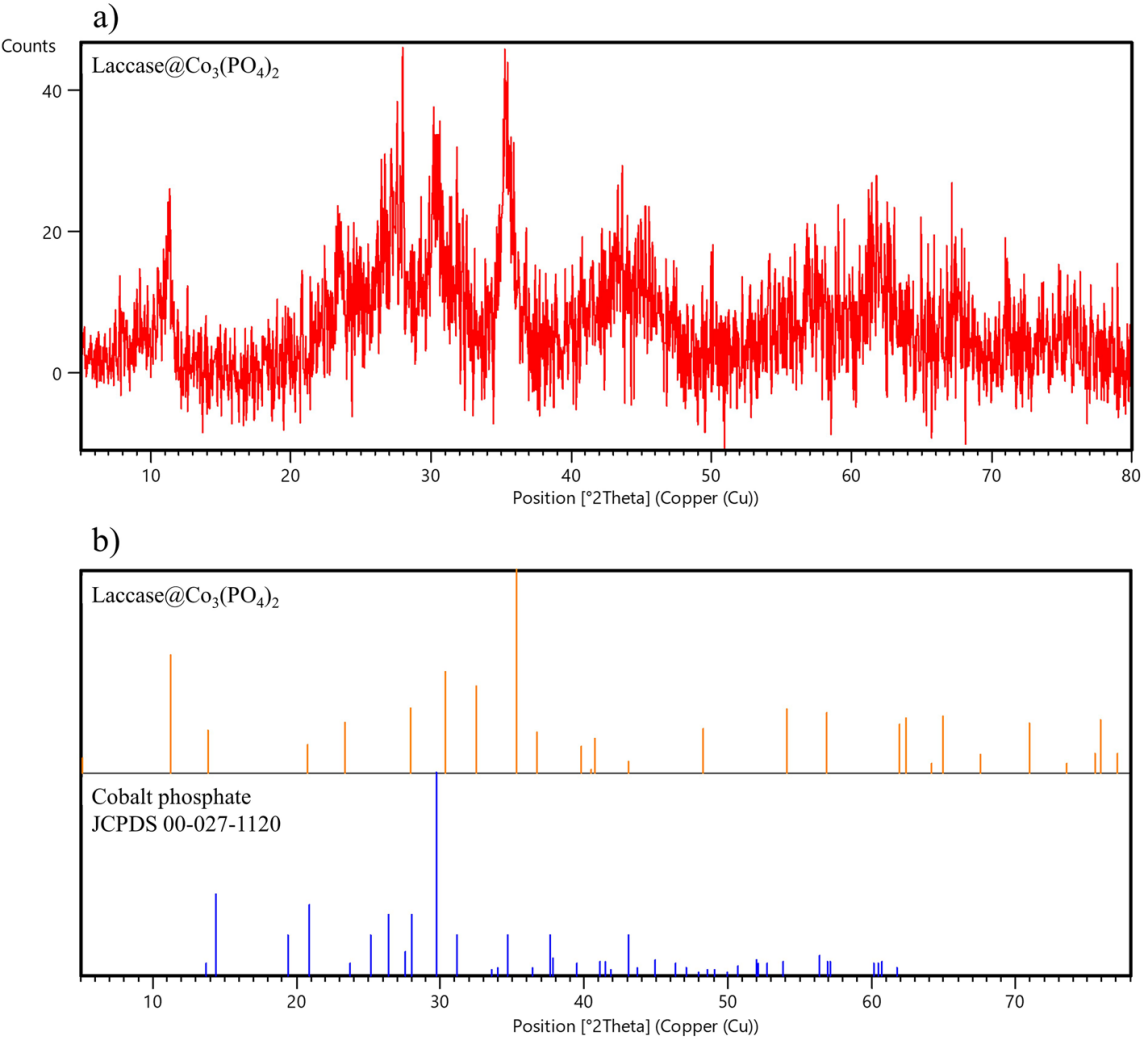
X-ray diffraction (XRD) analysis. X-ray spectrum of laccase@Co_3_(PO_4_)_2_•HNFs (**a**), XRD pattern of laccase@Co_3_(PO_4_)_2_•HNFs and the standard Co_3_(PO_4_)_2_ (JCPDS–00–027–1120) (**b**).

**Figure 4:**
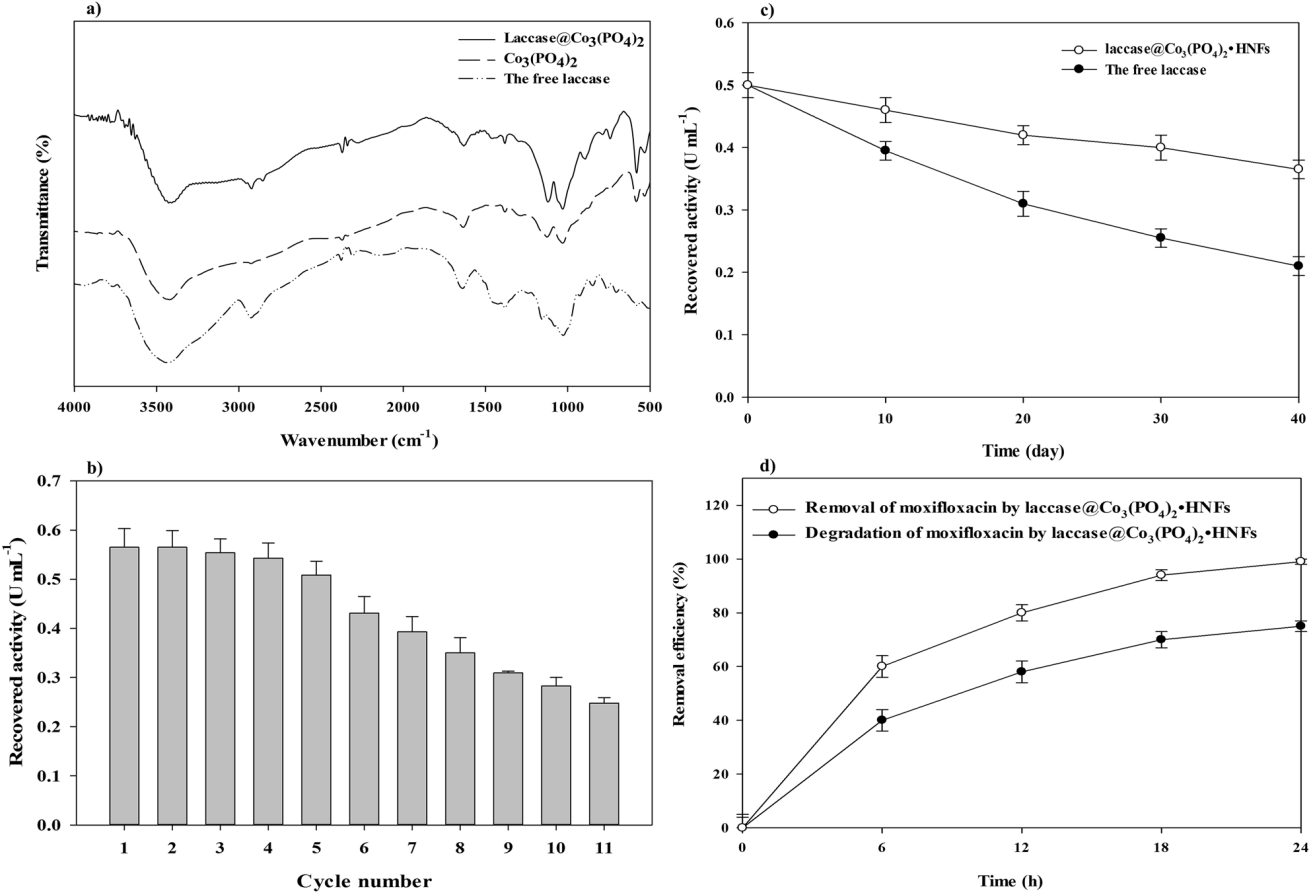
FT-IR analysis. The spectra of Fourier transform infrared (FTIR) spectroscopy of laccase@Co_3_(PO_4_)_2_•HNFs (solid line), Co_3_(PO_4_)_2_ (long dash line), and the free enzyme (dash-dot) (**a**). Reusability of laccase@Co_3_(PO_4_)_2_•HNFs (**b**). The activity of laccase@Co_3_(PO_4_)_2_•HNFs declined to under 50% of initial activity after 11 reusability cycles. Storage stability of laccase@Co_3_(PO_4_)_2_•HNFs and the free laccase after 40 days of storage at 4 °C (**c**). Bioremoval of moxifloxacin by laccase@Co_3_(PO_4_)_2_•HNFs and inactivated laccase@Co_3_(PO_4_)_2_•HNFs. The experiments were conducted at 40 °C in phosphate buffer (10 mM, pH 4.5) (**d**).

The flower-shaped morphology of the fabricated HNFs (Fig. 2a) was confirmed by SEM image. The size of laccase@Co_3_(PO_4_)_2_•HNFs ranged 0.5–3 µm and was assembled by interconnected nano-sheets resulting high surface-to-volume ratio (Fig. 2b). Cobalt phosphate nanoflowers (NFs) were also prepared through the same synthetic route without the incorporation of laccase in the reaction mixture. As shown in Fig. S1 and S2, the cobalt phosphate•NFs also exhibited flower-like morphology. Some of the previous studies reported that the flower-shaped morphology of HNFs is due to the presence of inorganic constituent(s)^44,45^. Also, it was reported that the 3D structure of the incorporated organic component could alter the morphology of HNFs^46^. However, inorganic copper phosphate was prepared by the same synthesis procedure, which exhibited the same flower-shaped morphology as protein@Cu_3_(PO_4_)_2_•HNFs^42,47–49^. All in all, it could be concluded that some metal phosphates have flower-like morphology^50^ that could be modified after hybridization with proteins. EDX analysis of laccase@Co_3_(PO_4_)_2_•HNFs and cobalt phosphate nanoflowers are exhibited in Fig. 2d and Fig. S4, respectively. The weight percentage of elements in both constructed HNFs and cobalt phosphate is summarized in Table S1. The presence of nitrogen atoms in the prepared HNFs confirms the immobilization of laccase into the support. Elemental map analysis (Fig. 2c) also provides information on the homogenous distribution of Co, P, N, and O atoms in the constructed HNFs. In order to identify the inorganic part of laccase@Co_3_(PO_4_)_2_•HNF, the XRD analysis was performed. The obtained peaks of laccase@Co_3_(PO_4_)_2_•HNF were in good agreement with the standard cobalt phosphate pattern (JCPDS 00–027-1120) (Fig. 3). Mean pore diameter, total pore volume, and specific surface area of the fabricated HNFs and Co_3_(PO_4_)_2_•NFs are summarized in Table S2, and corresponding BET plots are shown in Fig. S5. The high specific surface area (17.42 m^2^ g^−1^) is in agreement with the nano-sized width of petals in the SEM image of laccase@Co_3_(PO_4_)_2_. As shown, the specific surface area of Co_3_(PO_4_)_2_•NFs and the synthesized HNFs was almost identical. The presence of type IV isotherm in the BET plots is due to the mesoporous structure of the synthesized materials. As a rule of thumb, the higher surface area of the immobilized enzyme increases the accessibility of substrate to the enzyme and enhances the biocatalytic efficiency^51^. The same results were reported for laccase@Cu_3_(PO_4_)_2_•HNFs (10.2 m^2^ g^−1^) and GOx@Cu_3_(PO_4_)_2_•HNFs (17.9 m^2^ g^−1^)^52,53^. FTIR spectra of laccase, Co_3_(PO_4_)_2_, and laccase@Co_3_(PO_4_)_2_•HNFs are represented in Fig. 4a. As shown, characteristic vibrational frequencies in the region 725–1300 cm^−1^ are due to the presence of phosphate groups^54^. Peaks at 2980–3600 cm^−1^ were attributed to the presence of CH_2_ and CH_3_ groups in laccase@Co_3_(PO_4_)_2_•HNFs^54^. The broad band in 3200–3500 cm^−1^ corresponds to O–H stretching band^55^. A peak at 1640 cm^−1^ is attributed to the amide (I) band of laccase; however, it is overlapped with the absorption band of entrapped water in laccase@Co_3_(PO_4_)_2_•HNFs^56,57^.

### Structural studies

CD spectra were applied to elucidate the effect of immobilization on the secondary structure of laccase (Fig. S6). As summarized in Table 1, the conformation of the free native laccase was mainly composed of β-sheets (44.4%), β-turns (11.3%), and random coils (39.8%). Nevertheless, the amount of α-helix in the secondary structure of laccase increased remarkably (32%) after the immobilization of laccase in cobalt-based HNFs. The content of β-turns decreased slightly; however, β-sheets content (10.3%) of the laccase was significantly reduced. Laccase from *T. versicolor* is mainly composed of β-sheets, β-turns, and other structures, as reported previously^58,59^. Higher α-helix contents result in a more rigid protein structure, arising from the formation of a higher number of hydrogen bonds. Thus, by decreasing β-sheets and increasing α-helix contents, the laccase secondary structure became more rigid^60^. At first glance, it is not far-fetched that enzyme activity diminishes completely after such alteration in the protein structure. However, the immobilization of enzymes may significantly change the protein structure without loss of activity. For instance, by the preparation of cross-linked enzyme aggregates, the 3D structure of the enzyme is remarkably changed. Cross-linked laccase aggregated have been used for immobilization of the enzyme; meanwhile, the biocatalytic activity of the enzyme was preserved^61–63^. In order to verify the effect of any change in laccase tertiary structure, fluorescence spectroscopy was employed. The surrounding microenvironment highly influences the fluorescence properties of tryptophan; consequently, any change in the tryptophan microenvironment represents protein folding. As shown in Fig. S7, the fluorescence intensity of laccase@Co_3_(PO_4_)_2_•HNFs was lower than the free enzyme, which indicates the considerable change in the tertiary enzyme structure^64^. The results indicate that immobilization of laccase into cobalt phosphate HNFs increases the content of α-helix, which ultimately changes the tertiary structure of the protein. As shown in Fig. S8, the weight loss of laccase@Co_3_(PO_4_)_2_•HNFs was higher than the pure inorganic nanoflowers, which indicates that 5% of the weight of HNFs is composed of laccase. The total weight loss of laccase@Co_3_(PO_4_)_2_•HNFs upon decomposition was 28.02%. The initial weight loss of samples (6% at 100–160 °C) was due to the evaporation of crystal water entrapped in cobalt phosphate. At the second stage, the weight loss is attributed to the decomposition of immobilized laccase, which was 22%.

**Table 1:** Secondary structures content of the free laccase and laccase@Co_3_(PO_4_)_2_•HNFs.

Type of catalyst	α-Helix (%)	β-Sheet (%)	β-Turns (%)	Random coils (%)
The free laccase	5	44.4	11.3	39.4
Laccase@Co_3_(PO_4_)_2_•HNFs	32	10.3	6.5	51.2

*p < 0.05 vs. the free laccase.

### Enzyme activity of laccase@Co_3_(PO_4_)_2_•HNFs against temperatures and pH values

**Figure 5:**
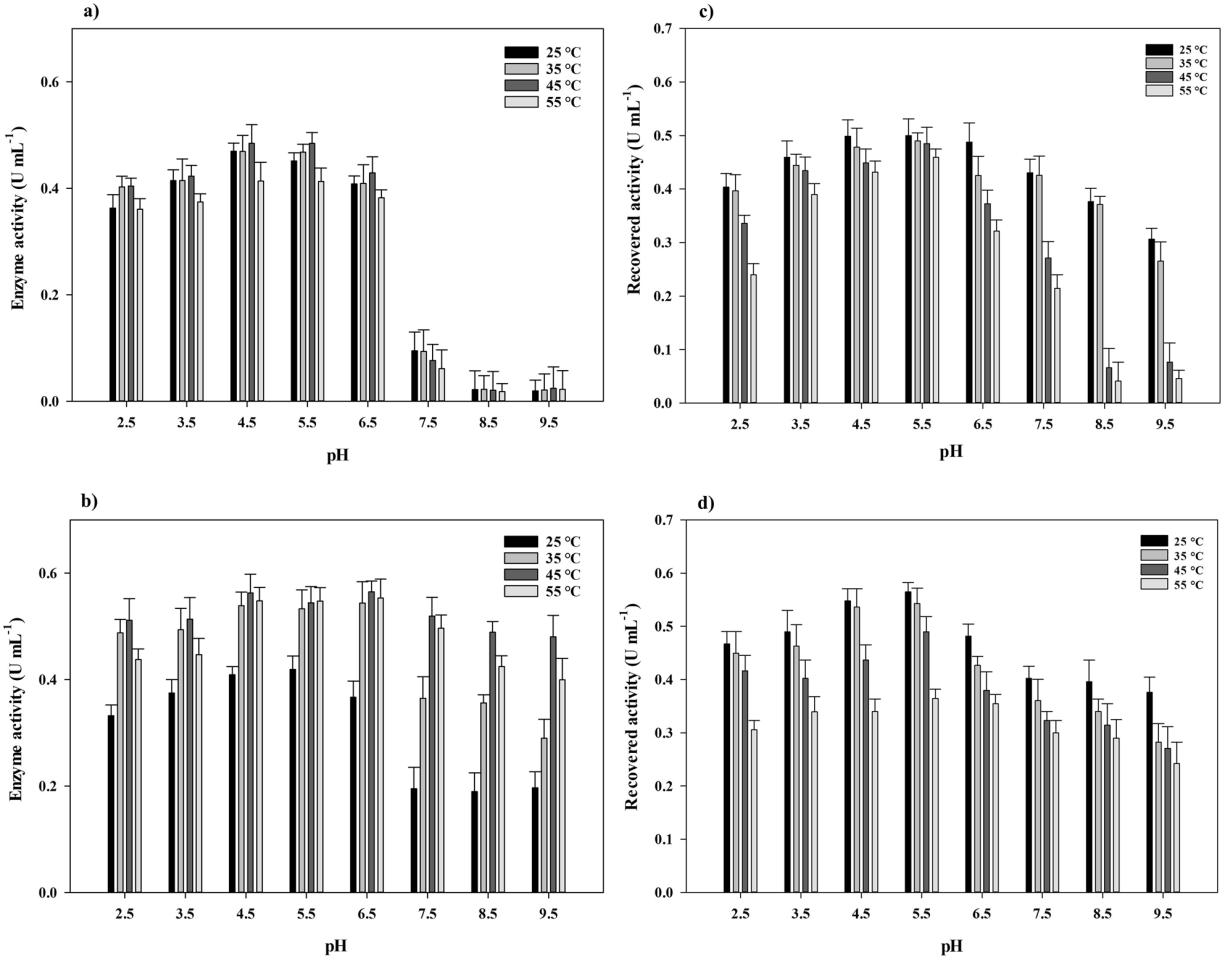
Stability and activity studies. Effect of pH and temperature on the activity (**a**) and stability of the free enzyme (**c**). Influence of pH and temperature on the activity (**b**) and stability of laccase@Co_3_(PO_4_)_2_•HNFs (**d**).

In order to determine the optimum conditions for the activity of laccase@Co_3_(PO_4_)_2_•HNFs, the enzyme activity was measured in a broad range of pH and temperature. As shown in Fig. 5a,b, the activity of the laccase@Co_3_(PO_4_)_2_•HNFs in basic pH values was remarkably higher than the free enzyme. However, maximum activity of both laccase@Co_3_(PO_4_)_2_•HNFs and the free enzyme was obtained at a pH range of 4.5–5.5 and 45 °C. Similar results were obtained by Patel et al.^65^ as the activity of cross-linked laccase@Cu_3_(PO_4_)_2_•HNFs at pH 4–7 was remarkably higher than the free enzyme. The suitable adaption of the enzyme with the surrounding microenvironment accounted for the increase in the activity of laccase at a wider pH range^65^. However, the following mechanisms were reported to be responsible for the improvement of laccase activity after immobilization into HNFs: (i) high surface area and porous structure of HNFs, (ii) allosteric effect of the inorganic component on enzyme active sites, and (iii) favorable conformation of entrapped enzyme^66,67^. It is well known that the immobilization of enzymes on supports with a high surface area is favorable due to the more enzyme loading^68^. Also, the high surface area of the immobilized enzyme results in the efficient mass transport of substrate to enzyme, therefore the activity of biocatalyst may be improved^69^. The allosteric effect of copper ions with laccase was reported to remarkably improve the enzymatic activity^70^. Also, the catalytic activity of α-amylase immobilized into CaHPO_4_ nanoflowers was improved due to the allosteric effect of Ca^2+^. However, the allosteric effect of Co^2+^ on laccase active sites was not previously reported. Therefore, it could be concluded that the high surface area of laccase@Co_3_(PO_4_)_2_•HNFs reduced mass transfer limitation, hence the substrate efficiently accessed the enzyme active site. It is believed that high temperatures and extreme acidic or basic pH reduce the activity and stability of the enzymes by induction of conformational changes^71^. However, it was shown that hybridization of nitrile hydratase (NHase) with cobalt phosphate complexes increased the stability of NHase against temperature^72^. Similarly, the activity of laccase was increased by 165% after incorporation into laccase@Cu_3_(PO_4_)_2_•HNFs^71^. Stable coordination of laccase with cobalt phosphate probably results in higher operational stability of the enzyme.

### Estimation of immobilization yield, efficacy, and kinetic parameters

Due to the improved enzymatic activity of the synthesized HNFs in basic pH values, kinetic properties of the immobilized and free laccase were evaluated at pH 4.5 and 7.4. Immobilization yield, efficiency, and kinetic properties of instantly-prepared laccase@Co_3_(PO_4_)_2_•HNFs and the free enzyme are summarized in Table 2. The IY (%) and IE (%) were 65.3 ± 7.6 and 110 ± 4.9, which were comparable with previous studies. It was reported that laccase was immobilized on the amino-functionalized metal–organic frameworks, which retained 93.8% of its initial activity. The enzyme loading (%) reached ≈10% and ≈70% after 1 h and 4 h of immobilization, respectively^73^. Laccase was also immobilized on the activated alumina pellets for decolorization of melanoidins. The immobilization process involved in the incubation of laccase with the support for 5 h, and the IY (%) and IE (%) were 87.5% and 97%, respectively^74^. Laccase-lysine@Cu_3_(PO_4_)_2_•HNFs were prepared by the incubation of the synthesis mixture for 72 h, and the IY (%) and activity recovery were 71% and 270%, respectively. It was reported that the prepared HNFs exhibited dual oxidase and peroxidase activity. The remarkable enhancement of the enzymatic activity after the immobilization accounted for favorable conformational change of laccase due to the presence of copper ions and lysine in the structure of HNFs^70^.

**Table 2:** Kinetic parameters of the free laccase and laccase@Co_3_(PO_4_)_2_•HNFs.

Parameter	The free laccase		Laccase@Co_3_(PO_4_)_2_•HNFs	
	pH		pH	
	4.5	7.4	4.5	7.4
*V*_max_ (µmol min^−1^)	8.4 ± 0.4	6.2 ± 0.2	4.7 ± 0.2	6.6 ± 0.3
*K*_m_ (µM)	147.9 ± 8.5	118.5 ± 5.3	85.4 ± 4.3	110.7 ± 4.6
*K*_cat_ × 10^−2^ (S^−1^)	6.5 ± 0.2	4.8 ± 0.2	3.6 ± 0.1	5.1 ± 0.1
Catalytic efficiency × 10^−4^ (*K*_cat_/*K*_m_)	4.4 ± 0.3	4.0 ± 0.2	4.2 ± 0.3	4.6 ± 0.4
Immobilization yield (%)	–	–	65.3 ± 7.6	65.3 ± 7.6
Immobilization efficacy (%)	–	–	86 ± 5.3	110 ± 4.9

Immobilization efficiency, *V*_max_, and *K*_m_ of the biocatalysts were different in basic and acidic conditions. The *V*_max_ value of the laccase@Co_3_(PO_4_)_2_•HNFs at pH 7.4 was 40% higher than pH 4.5; however, the maximum velocity of the free enzyme declined 21.2% at pH 7.4. Likewise, the efficiency of the immobilization procedure was increased by 25% at pH 7.4. The results indicated that *K*_cat_ of laccase@Co_3_(PO_4_)_2_•HNFs was higher than the free enzyme at pH 7.4; while, the free enzyme exhibited superior *K*_cat_ over laccase@Co_3_(PO_4_)_2_•HNFs at pH 4.5. These results are in alignment with the effect of pH on the activity of both free and immobilized laccase. The Michaelis constant (*K*_m_) indicates the affinity of the enzyme toward the substrate, and a low *K*_m_ value indicates the high affinity of the enzyme toward the substrate in both pH values. The improved accessibility of laccase@Co_3_(PO_4_)_2_•HNFs to the substrate (compared to the free enzyme) could be attributed to the high surface area of the flower-shaped structures that reduces the mass transfer limitations^75^. Normally, *K*_m_, *V*_max_, and the optimum conditions for reaching the highest catalytic efficiency enzymes may alter after immobilization^76^. The maximum activity of fungal laccases has been reported to be obtainable at a pH range of 3–5^77^. One of their main limitations is an insufficient activity in neutral and alkaline conditions despite the high redox potential of fungal laccases (490–790 mV). In this regard, pH-tolerant laccases are desirable for industrial and environmental purposes^78^. Hybridization of laccase with cobalt phosphate nanocrystals enhanced the tolerance of laccase from *T. versicolor* toward alkaline conditions, which could improve their desirability for industrial and environmental applications^79^.

### Reusability and stability of laccase@Co_3_(PO_4_)_2_•HNFs

Reusability is an important feature of biocatalysts that reduces their utilization cost, especially on the industrial scale. However, in the present study, measurement of the reusability for laccase@Co_3_(PO_4_)_2_•HNFs provided evidence on the successful immobilization of laccase into the matrix of cobalt phosphate. In order to evaluate the reusability of laccase@Co_3_(PO_4_)_2_•HNFs, the enzyme activity was consecutively measured by ABTS as the substrate. As shown in Fig. 4b, after 4 consecutive reuse cycles, the activity of the laccase@Co_3_(PO_4_)_2_•HNFs did not significantly change. However, the enzyme activity declined to 50% of its initial value after 10 cycle reuses. The results indicate that laccase@Co_3_(PO_4_)_2_•HNFs exhibited remarkable reusability, and the enzyme was successfully immobilized in the matrix of cobalt phosphate. Laccase-based HNFs were exhibited promising reusability; for instance, laccase and lysin-copper HNFs retained 60% of initial activity after 6 cycles of reusability^81^. In another study, laccase-copper HNFs retained 50% of the initial activity after 10 reusability runs; however, the glutaraldehyde-treated HNFs retained almost 100% of their initial activity under the same conditions^65^. As shown in Fig. 5c,d, the stability of laccase@Co_3_(PO_4_)_2_•HNFs against elevated temperatures and basic pH values was higher than the free laccase. Laccase@Co_3_(PO_4_)_2_•HNFs retained 50% of their activity after incubation at pH 9.5 for 3 h, where the free laccase almost lost all of its activity. It should be noted that the stability of laccase@Co_3_(PO_4_)_2_•HNFs was lower than the free enzyme in acidic pH values due to the solubility of cobalt phosphate in acidic pH^25^. No data is available for comparison. As mentioned in 3.3, the α-helix content of laccase was increased after hybridization with cobalt phosphate. Copper ions of laccase, which are involved in the catalytic activity of the enzyme, are present in β-sheets, which are mainly composed of conserved polar amino acids^82^. The stability and activity of laccase have been reported to be increased when the content of α-helix increased and β-sheets decreased^20,60,83^. A more rigid protein structure aligned with increasing in α-helix content could explain the higher stability of laccase HNFs than the free enzyme^20^. However, the stability of enzymes immobilized on cobalt-based supports was enhanced^84^. For instance, laccase was immobilized on the Co^2+^ and Cu^2+^-based MOFs, which resulted in an improvement of its enzymatic activity. The laccase-Co^2+^ and laccase-Cu^2+^ MOFs retained 66% and 58% of their initial activity after 4 weeks of storage at room temperature, indicating the superior stabilization of laccase by Co^2+^-based MOF^85^. The thermal stability of laccase was improved after incorporation into laccase@Cu_3_(PO_4_)_2_•HNFs^71^. In another study, laccase was immobilized on the CoFe_2_O_4_, Fe_3_O_4_, and NiFe_2_O_4_ nanoparticles. Laccase-CoFe_2_O_4_, laccase-Fe_3_O_4_, laccase-NiFe_2_O_4_, and the free laccase retained **≈**80%, **≈**70%, **≈**40%, and 0% of their initial activity, respectively, after 30 days of incubation at 25°C^86^. It was reported that 43% and 22% of laccase@Cu_3_(PO_4_)_2_•HNFs and the free enzyme, respectively, were retained after 30 min incubation at 80 °C. It was reported that conformational stability and stiffness of the immobilized laccase were responsible for the stabilization of laccase. Graphene oxide-laccase HNFs were synthesized for efficient dye removal of crystal violet (CV) and neutral red (NR). After 60 min of incubation at 70 °C, the free and immobilized laccase preserved 57% and 71% of their initial activity, respectively^75^. Immobilization of laccase into laccase@Co_3_(PO_4_)_2_•HNFs increased the storage stability of the enzyme. As shown in Fig. 4c, after 40 days of storage at 4 °C, the prepared HNFs lost 20% of the initial activity, while the free enzyme lost 58% of the initial activity.

### Bioremoval of moxifloxacin

As shown in Fig. 4d, laccase@Co_3_(PO_4_)_2_•HNFs were able to remove moxifloxacin significantly. The antibiotic was nearly completely (99%) eliminated by the fabricated HNFs from the reaction mixture by both degradation and adsorption mechanisms. In order to specifically determine the adsorption of moxifloxacin on the constructed HNFs, the antibiotic was treated by deactivated immobilized laccase. Therefore, the amount of moxifloxacin removal by deactivated laccase@Co_3_(PO_4_)_2_•HNFs was accounted for the adsorption mechanism. Adsorption on the surface of HNFs was responsible for 24% of moxifloxacin removal after 18 h of treatment, and the maximum adsorptive capacity of deactivated laccase@Co_3_(PO_4_)_2_•HNFs for this antibiotic was found to be 0.8 mg g^−1^. In a recent study, doxorubicin was removed from human urine using magnetic nanoflowers of copper phosphate. It was reported that by complexing with the aglycone ring and the sugar moiety of doxorubicin, metal ions such as Cu(II), Fe(II or III), and Mn(II) could bond to this antitumor antibiotic^49^. It was previously reported that metal^2+^ ions could form complexes with quinolone-like antibiotics through a complexation mechanism with 4-oxo and adjacent carboxyl groups of quinolone moiety. Therefore, the complexation of quinolone-like antibiotics with metal^2+^ ions could be used for their adsorption^87,88^. As shown by Wu et al. ^89^, montmorillonite and kaolinite adsorbed nalidixic acid through the complexation with C-3 carboxyl (OH) group and C-4 oxo group of this synthetic fluoroquinolone. The maximum adsorption capacity of montmorillonite and kaolinite for nalidixic acid was 24 and 1.03 mg g^−1^, respectively^89^.

As shown, the degradation of moxifloxacin by laccase@Co_3_(PO_4_)_2_•HNFs reached a plateau after 18 h of treatment and accounted for 75% of its removal. The elimination of moxifloxacin by laccase has not been reported previously; however, other quinolone-like antibiotics such as ciprofloxacin and ofloxacin were efficiently removed by laccase^90–92^. In this study, moxifloxacin was removed by laccase@Co_3_(PO_4_)_2_•HNFs in the presence of HBT, a laccase mediator. Several types of pharmaceuticals cannot be degraded by laccases, such as quinolone-like antibiotics, due to their high redox potential (*E*°)^93^. Laccase/mediator systems (LMSs) have been applied to overcome this limitation by increasing the oxidation potential of laccase toward high *E*° of organic compounds^93^. The free radicals generated through the oxidation of mediators by laccase can efficiently oxidize a wide variety of organic substances. Indeed, mediators act as redox shuttles between laccase active sites and substrates with high redox potential^94^. With the addition of HBT, 40% and 75% of moxifloxacin was degraded after 6 and 24 h treatment, respectively. The same results were reported for the degradation of iso-butylparaben (iso-BP) and n-butylparaben (*n*-BP) by laccase/HBT oxidation system. As laccase could not remove iso-BP and n-BP, they were almost completely removed by laccase/HBT (2 mM) system^95^. In another study, laccase from *Coriolopsis gallica* UAMH8260 was utilized for the degradation of some textile dyes. It was reported that the enzyme could decolorize 13 synthetic textile dyes; however, the laccase/HBT (1 mM) system could efficiently remove 26 textile dyes^96^.

### Identification of degradation products

**Figure 6:**
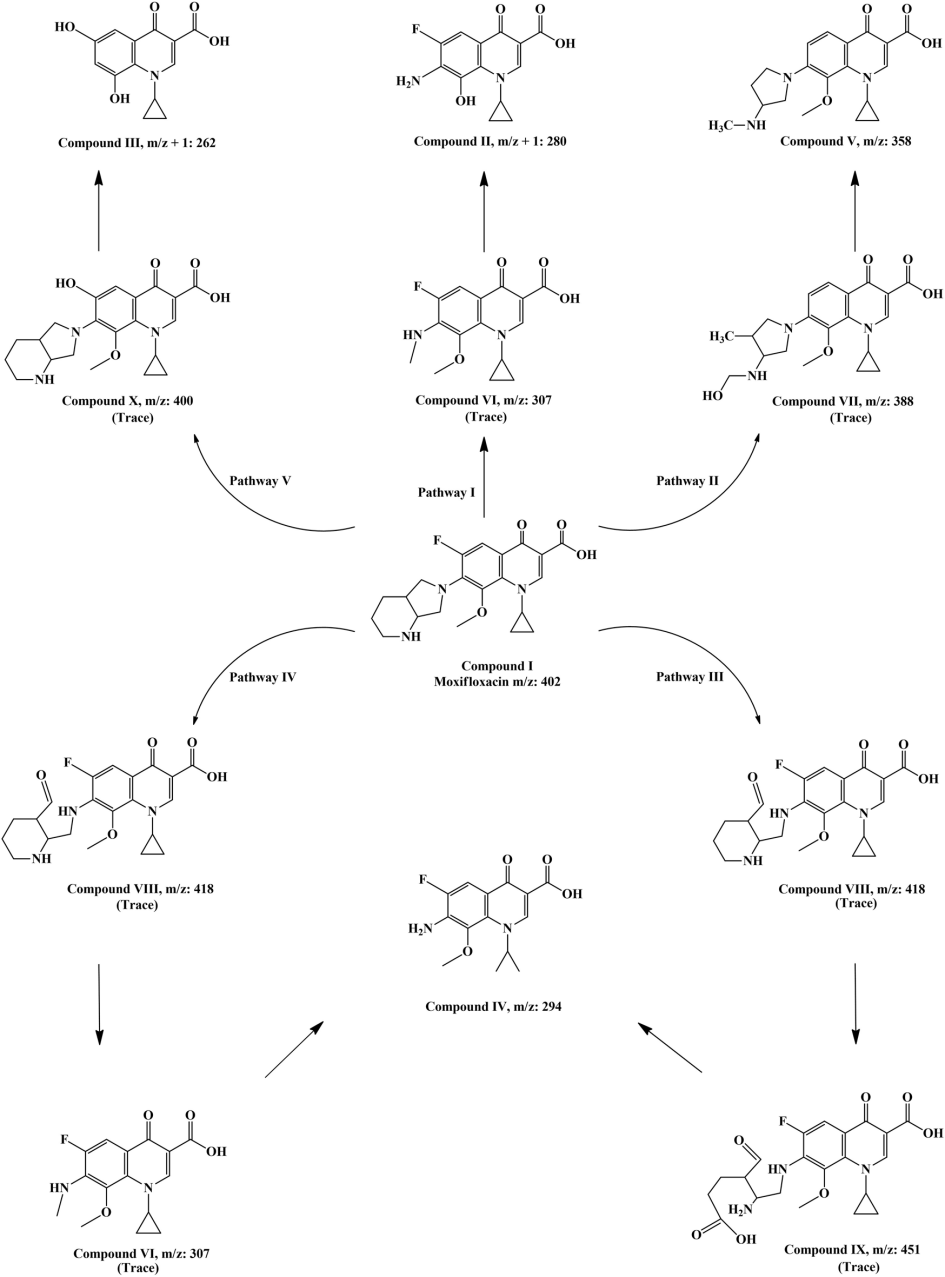
Mechanism of moxifloxacin degradation. Proposed moxifloxacin biotransformation pathways by laccase@Co_3_(PO_4_)_2_•HNFs.

After the bioremoval procedure, the mixture of moxifloxacin biotransformed products was subjected to LC–MS analysis. The main by-products, their retention times, and corresponding mass to charge ration (m/z) are presented in Fig. S9. Based on the LC–MS results, five plausible pathways for the degradation of moxifloxacin are proposed (Fig. 6). Interestingly, the biocatalyst replaced the fluorine atoms from moxifloxacin, which is responsible for the low biodegradability of FQs, with hydroxyl groups (compound III; m/z + 1 = 262, R_t_ = 6 min)^90^. As shown, ring-opening of piperazine moiety and further demethylation reactions were responsible for appearing compound VI (m/z = 307, trace) and compound II (m/z + 1 = 280, R_t_ = 3.9 min), respectively (pathway I). In pathway II, moxifloxacin underwent defluorination and cleavage of the piperazine moiety (compound VII; m/z = 388, trace) and further transformed to compound V (m/z = 358, R_t_ = 9.8 min) after dihydroxylation and demethylation^97^. In the 3rd pathway, by oxidation and opening of piperazine ring, compound VIII (m/z = 418, trace) was formed, which finally converted to compound IV (m/z = 294, R_t_ = 8.7 min) after subsequent oxidation steps. In the next pathway, compound VIII (m/z = 418, trace) was formed, and by complete cleavage of piperazine group, compound I was transformed to compound IX (m/z = 307, trace) that was transformed to compound IV (m/z = 294, R_t_ = 8.7 min) after demethylation and cracking of cyclopropane ring (pathway IV). In the final proposed mechanism, moxifloxacin fluor atom was substituted with OH (compound X, m/z = 400, trace) and further, by loss of piperazine group and demethylation, converted to compound III (m/z + 1 = 262, R_t_ = 6 min) (pathway V).

### Toxicity of the biodegradation products

Diminishing toxicity of pollutants is not always guaranteed by degradation procedures; even there are reports on an increase in toxicity of pollutants after degradation^98,99^. In order to examine the efficiency of the synthesized HNFs, the toxicity of moxifloxacin and its metabolites against four bacterial strains was evaluated. As shown in Table S3, the toxicity of the degradation products was effectively decreased after treatment with laccase@Co_3_(PO_4_)_2_•HNFs. Treatment of moxifloxacin with the constructed HNFs reduced the GI% for *P*. *aeruginosa* by 67%. GI reduction (%) for *S*. *aureus, S. epidermidis,* and *E*. *coli* was 24.6%, 48.7%, and 12%, respectively. Some of the FQs degradation products exhibited antimicrobial activity. Due to the enhancement of DNA gyrase activity by fluorine, the defluorination of moxifloxacin significantly reduced its biological activity. Also, most of the identified degradation products possess lower hydrophobicity, which may limit their diffusion through the cell membrane^97,100,101^.

## Conclusion

This study reported a facile approach for the instant synthesis of HNFs termed the "concentrated method". Contrary to previously-reported synthesis procedures, high concentrations of phosphate and cobalt ions were incorporated for the synthesis of HNFs. The formation of primary nucleation sites (protein-inorganic nanocrystals) and their anisotropic growth were the main steps involved in the synthesis of HNFs by this method. Rapid fabrication of HNFs was achieved by increasing the number of primary nanocrystals, which improved their collision rate and accelerated the growth of HNFs. Therefore, the synthesis of laccase@Co_3_(PO_4_)_2_•HNFs did not involve prolonged incubation times (12–72 h) and harsh conditions such as ultrasonication and vortexing. Catalytic efficiency, kinetic parameters, and stability of laccase were enhanced after the enzyme immobilization into HNFs. However, the immobilization yield was not very high (65.3%) compared to the previous published works, this may reduce the cost-effectiveness of the immobilization method. Based on CD and fluorimetry spectra, higher α-helix content of laccase@Co_3_(PO_4_)_2_•HNFs than the free enzyme is responsible for the enhanced stability of the constructed HNFs. The ability of the immobilized laccase to remove moxifloxacin was firstly presented through this study. The antibiotic was removed by both adsorption and biodegradation mechanisms, and the toxicity of degradation products against two G^+^ and two G^−^ bacteria was lower than the parent fluoroquinolone. However, the toxicity of the degradation products against *S. aureus* (18.5%) and *E. coli* (8.8%) was minorly reduced. The described method for the synthesis of laccase@Co_3_(PO_4_)_2_•HNFs could be used for the synthesis of a wide range of HNFs using various organic and inorganic components. Exploring the formation of laccase-based HNFs with different metals will shed light on the interaction of laccase with solid-state metal phosphates. Also, the present knowledge of the formation mechanism of HNFs is currently limited and further studies are needed for exploring new methods for the synthesis of laccase-based HNFs.

## Supplementary Information

Supplementary Information.

## Data Availability

All data generated or analysed during this study are included in this published article (and its Supplementary Information files).
