# Supplementary material for: Instantaneous synthesis and full characterization of organic–inorganic laccase-cobalt phosphate hybrid nanoflowers
Source: Sci Rep. 2022 Jun 3;12:9297. doi: 10.1038/s41598-022-13490-w (PMC9165545; doi:10.1038/s41598-022-13490-w)
Supplement: Supplementary file 1 — Supplementary Information. [file 41598_2022_13490_MOESM1_ESM.docx]

**Supplementary Material**

**Instantaneous synthesis and full characterization of organic-inorganic laccase-cobalt phosphate hybrid nanoflowers**

Khashayar Vojdanitalab^a,b,#^, Hossein Jafari-Nodoushan^a,#^, Somayeh Mojtabavi^a^, Mahtab Shokri^a,b^, Hoda Jahandar^b^, and Mohammad Ali Faramarzi^a,*^

*^a^ Department of Pharmaceutical Biotechnology, Faculty of Pharmacy & Biotechnology Research Center, Tehran University of Medical Sciences, P.O. Box 14155*−*6451, Tehran 1417614411, Iran*

*^b^ Pharmaceutical Sciences Research Center, Tehran Medical Sciences Branch, Islamic Azad University, Tehran, Iran*

-------------------------------------------------

*Corresponding author: M.A. Faramarzi, Telefax: +98-21-66954712, E-mail: faramarz@tums.ac.ir

^#^K. Vojdanitalab and H. Jafari-Nodoushan contributed equally as first author.


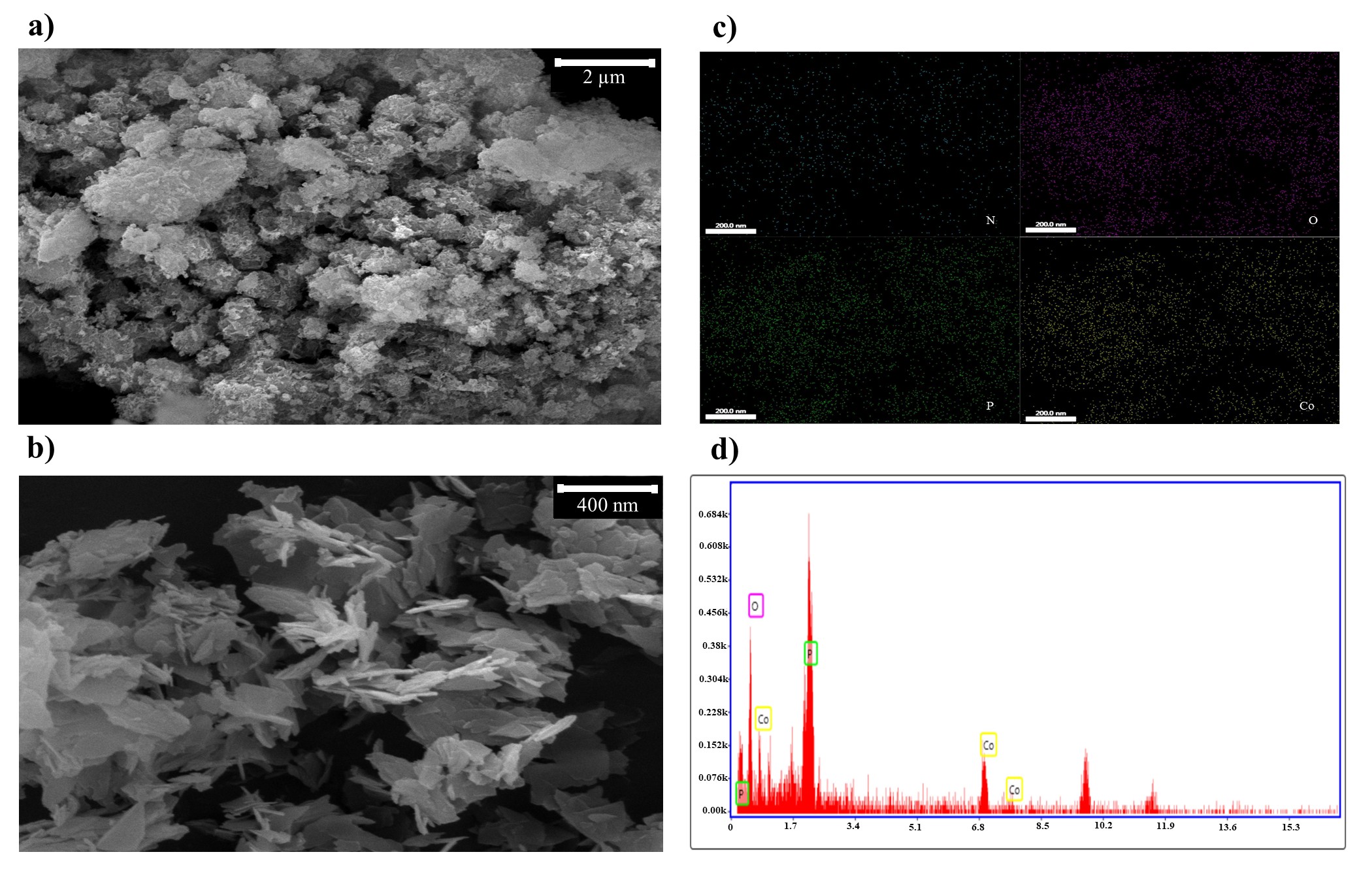


**Fig. S1.** Scanning electron microscopy (SEM) image of the constructed Co_3_(PO_4_)_2_•NFs. The spherical flower-shaped nanostructures are formed by the “concentrated method.”


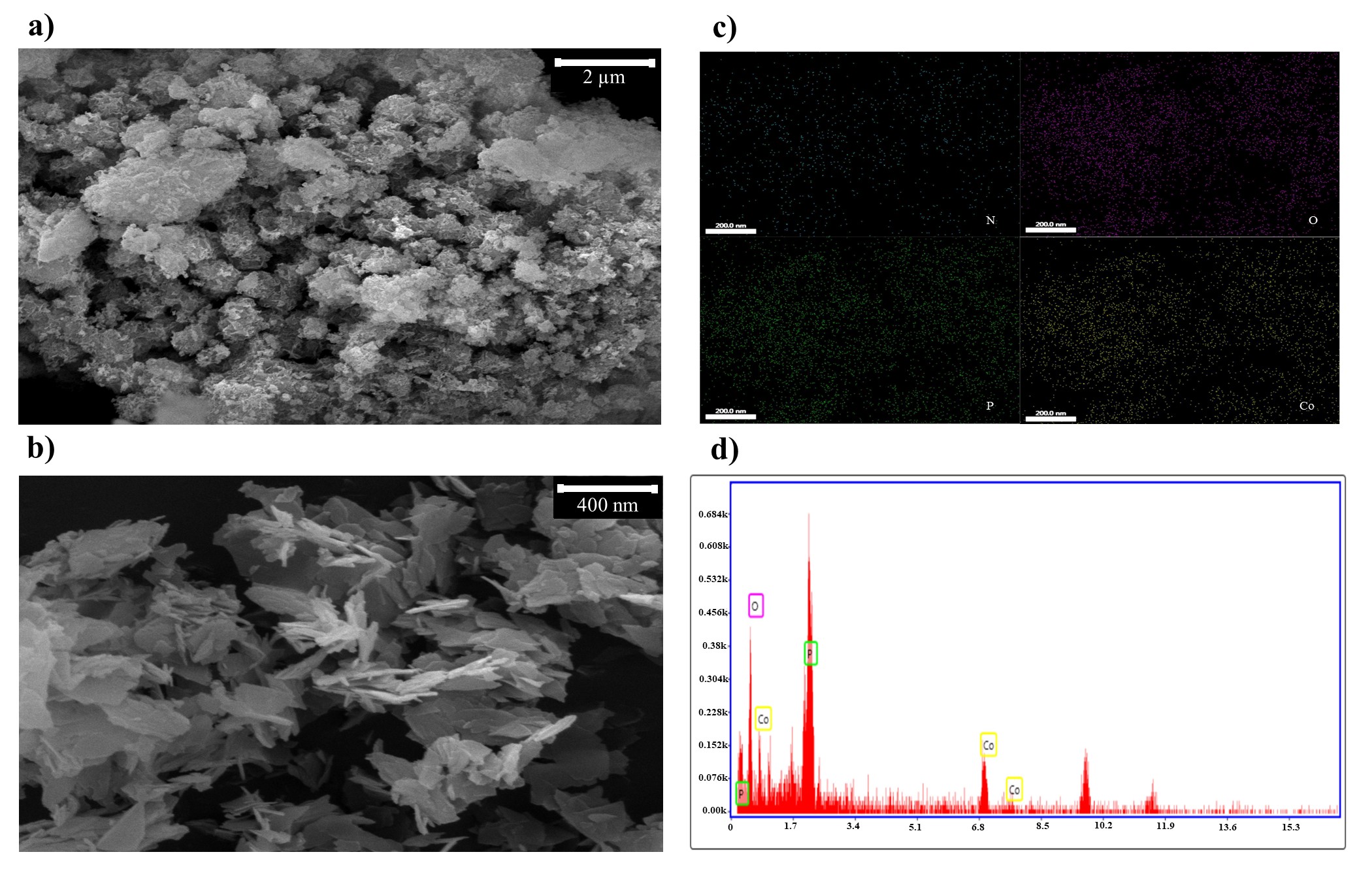


**Fig. S2.** Scanning electron microscopy (SEM) image of the constructed Co_3_(PO_4_)_2_•NFs revealed that the flower-shaped nanostructures were composed of ultrathin nanosheets.


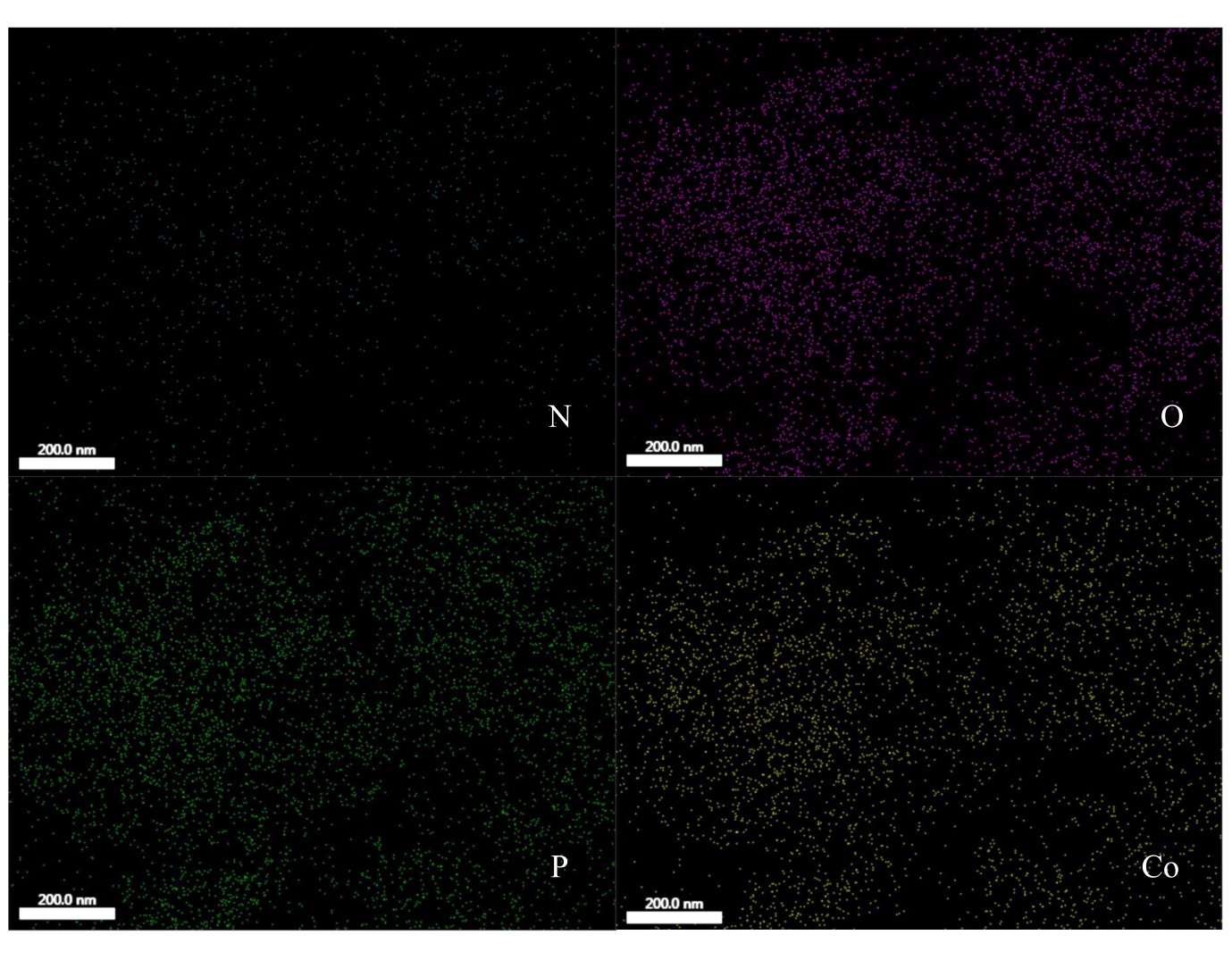


**Fig. S3.** Elemental map analysis of Co_3_(PO_4_)_2_•NFs that mostly composed of P, Co, and O elements.


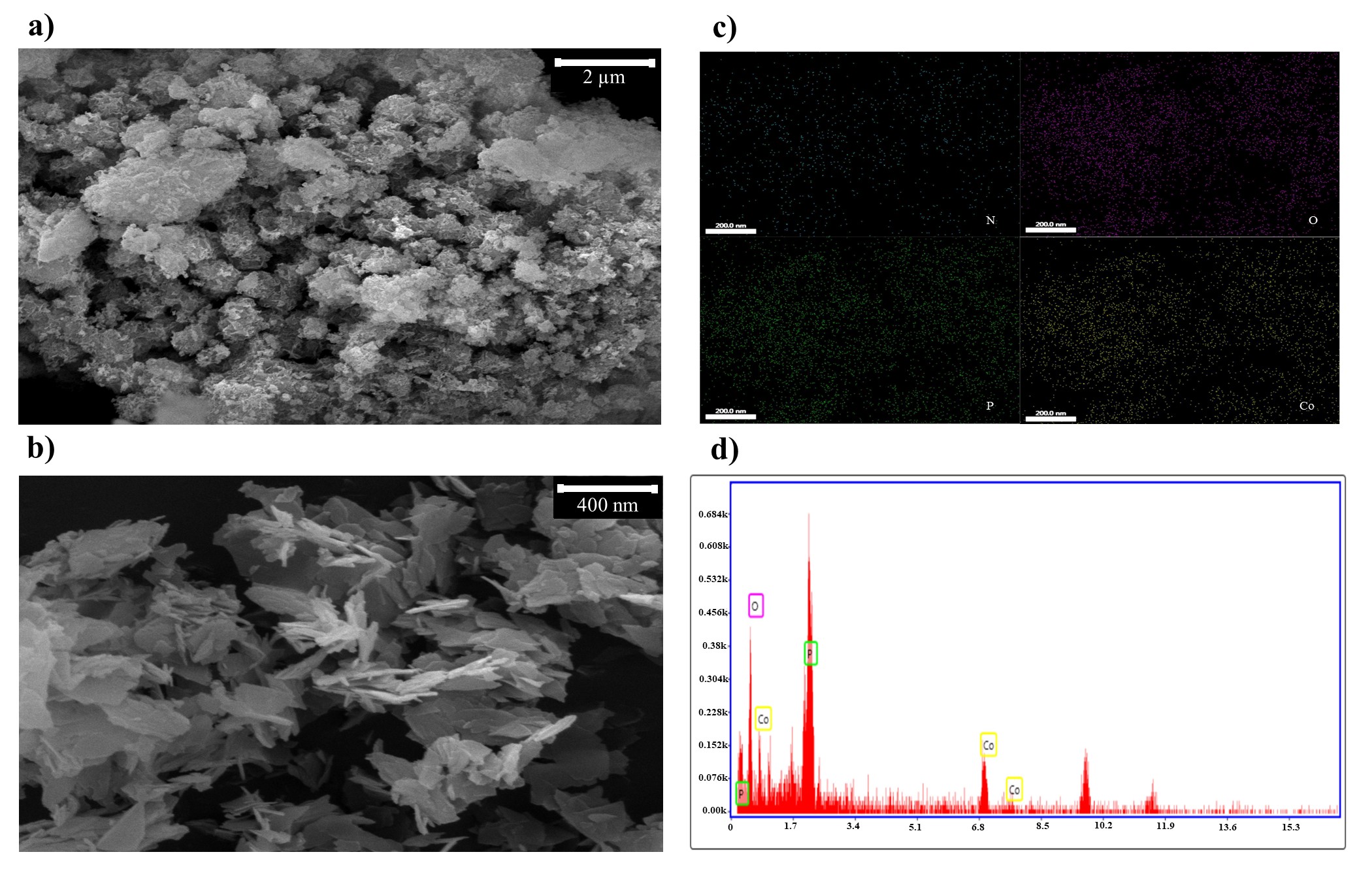


**Fig. S4.** Energy dispersive X-Ray spectroscopy (EDX) analysis of the constructed Co_3_(PO_4_)_2_•NFs.


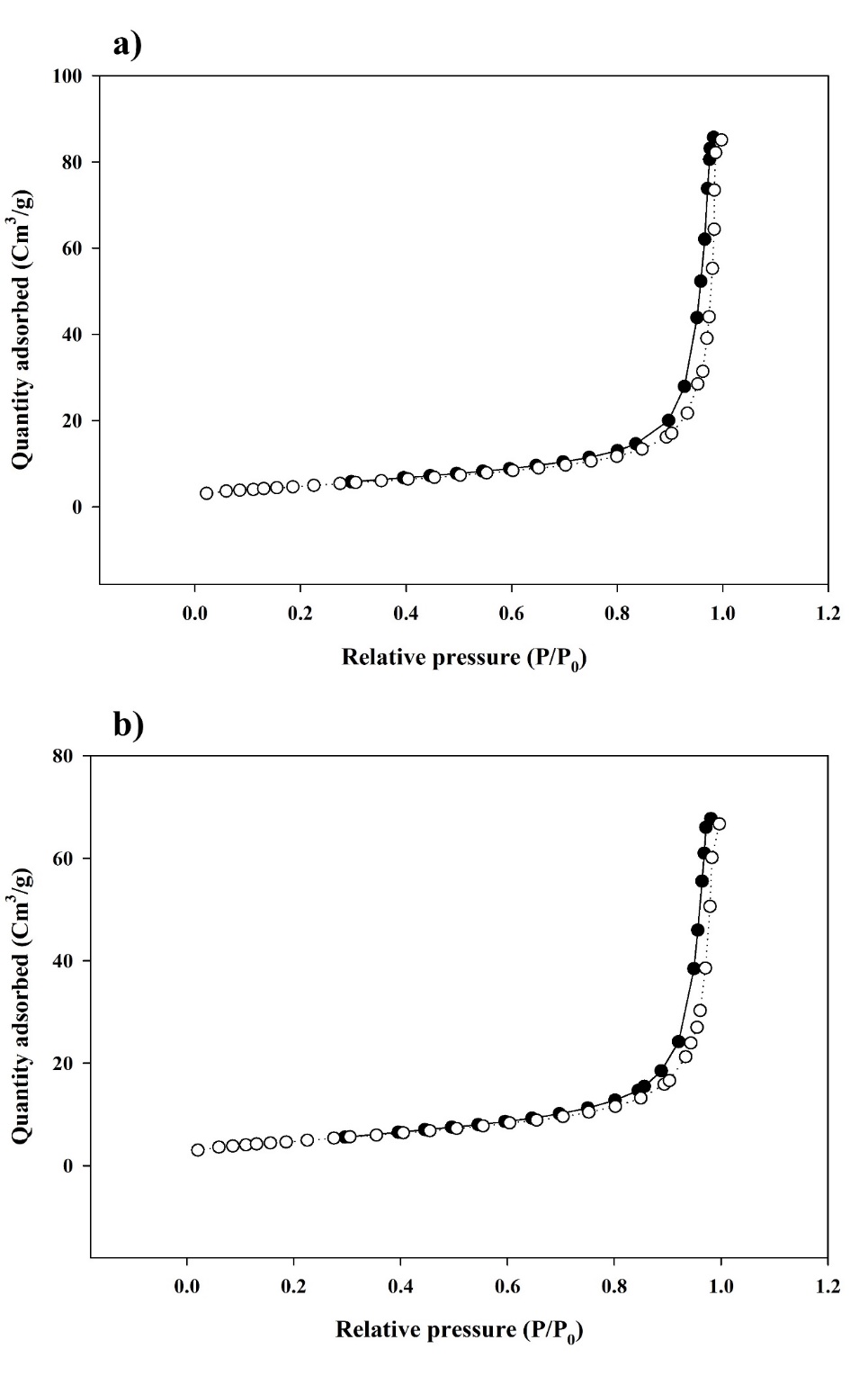


**Fig. S5.** Brunauer-Emmett-Teller (BET) plot of laccase@Co_3_(PO_4_)_2_•HNFs (a) and Co_3_(PO_4_)_2_•NFs (b). Both materials exhibited type IV isotherm with a narrow hysteresis loop, which is attributed to the mesoporous structure of the synthesized HNFs and NFs.


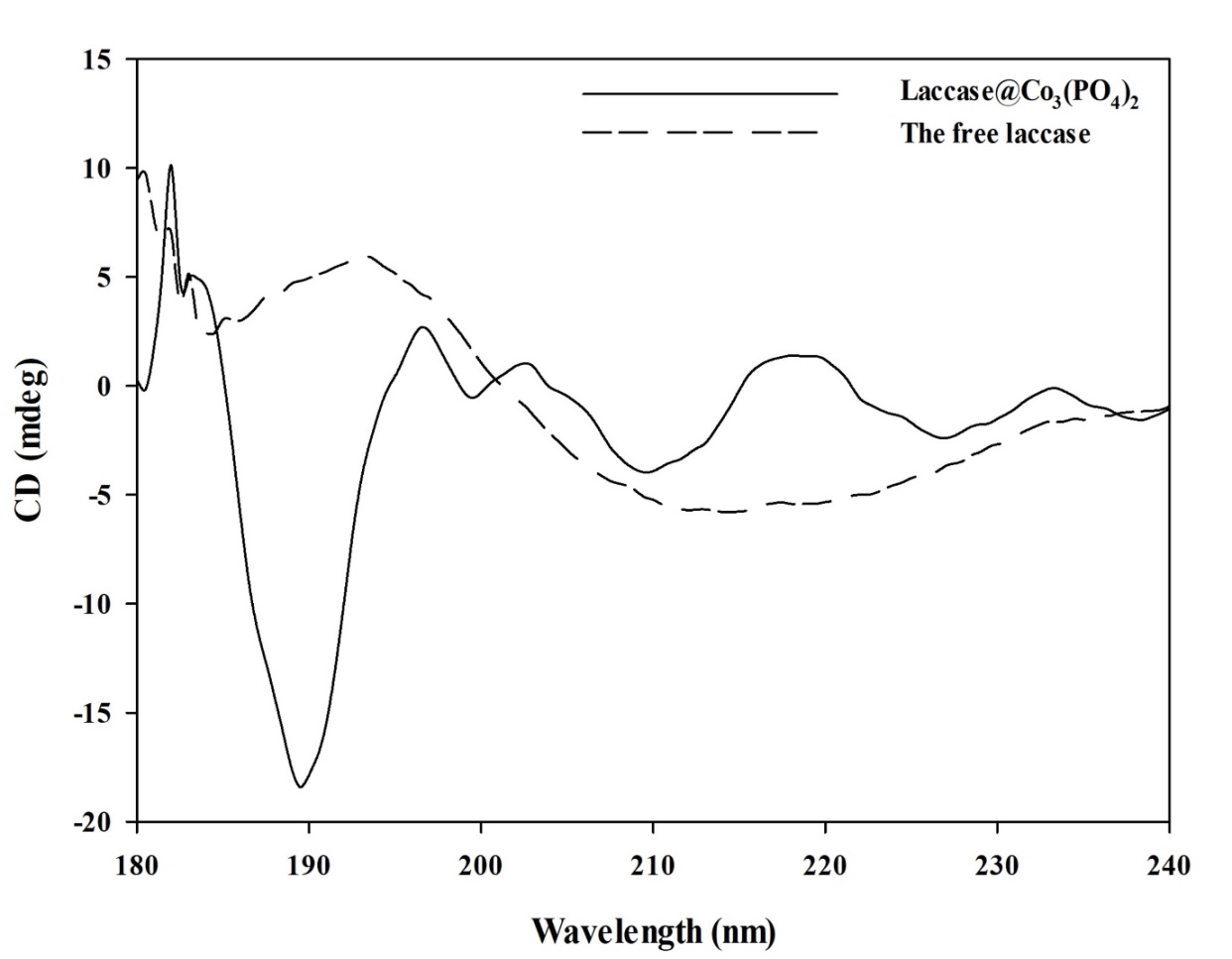


**Figure S6.** Circular dichroism (CD) spectra of laccase@Co_3_(PO_4_)_2_•HNFs and the free enzyme recorded at 180–240 nm. Coordination of laccase with cobalt phosphate significantly altered the secondary structure of the enzyme.


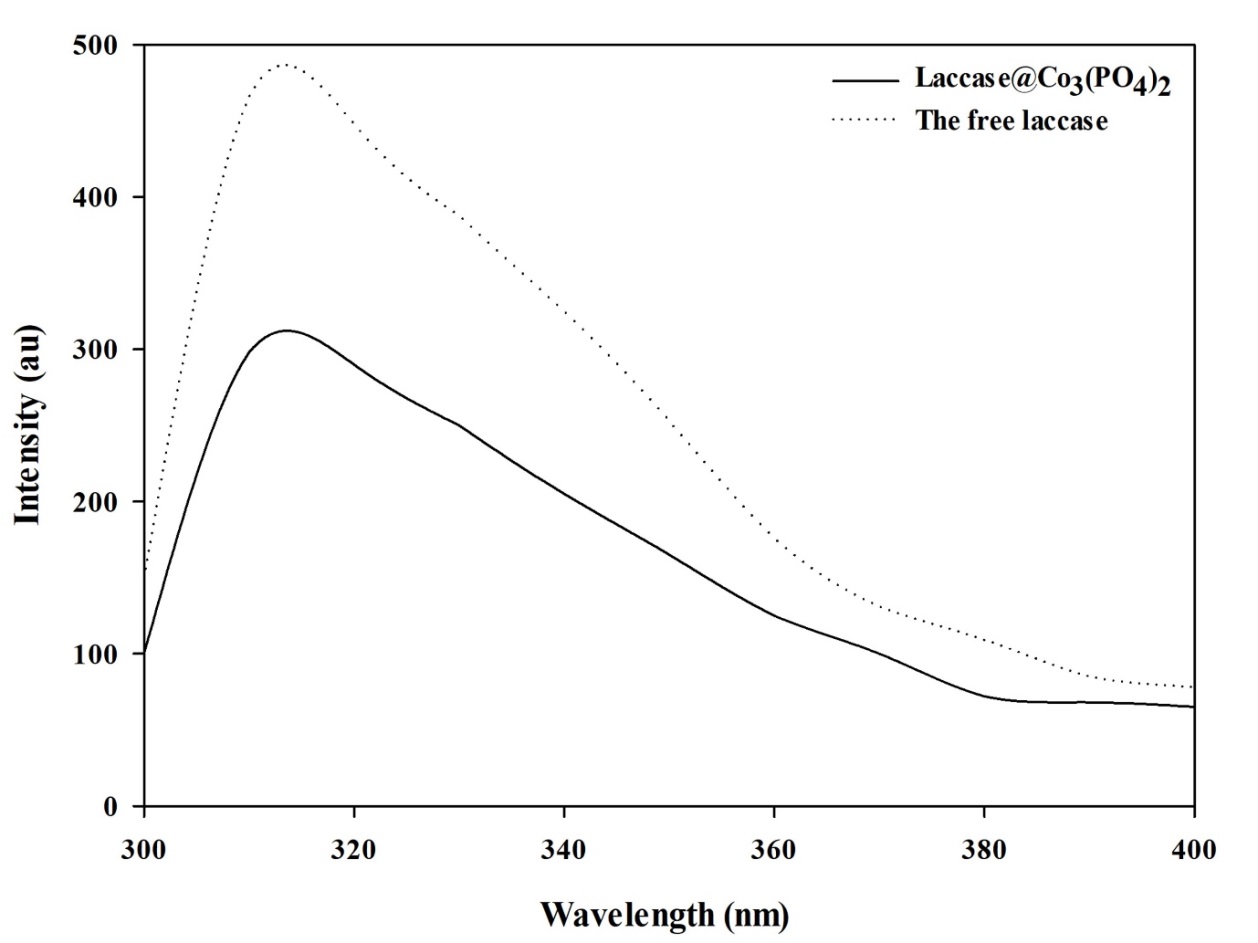


**Figure S7.** Tryptophan fluorescence intensity of laccase@Co_3_(PO_4_)_2_•HNFs and the free enzyme for estimation of the protein tertiary structure.


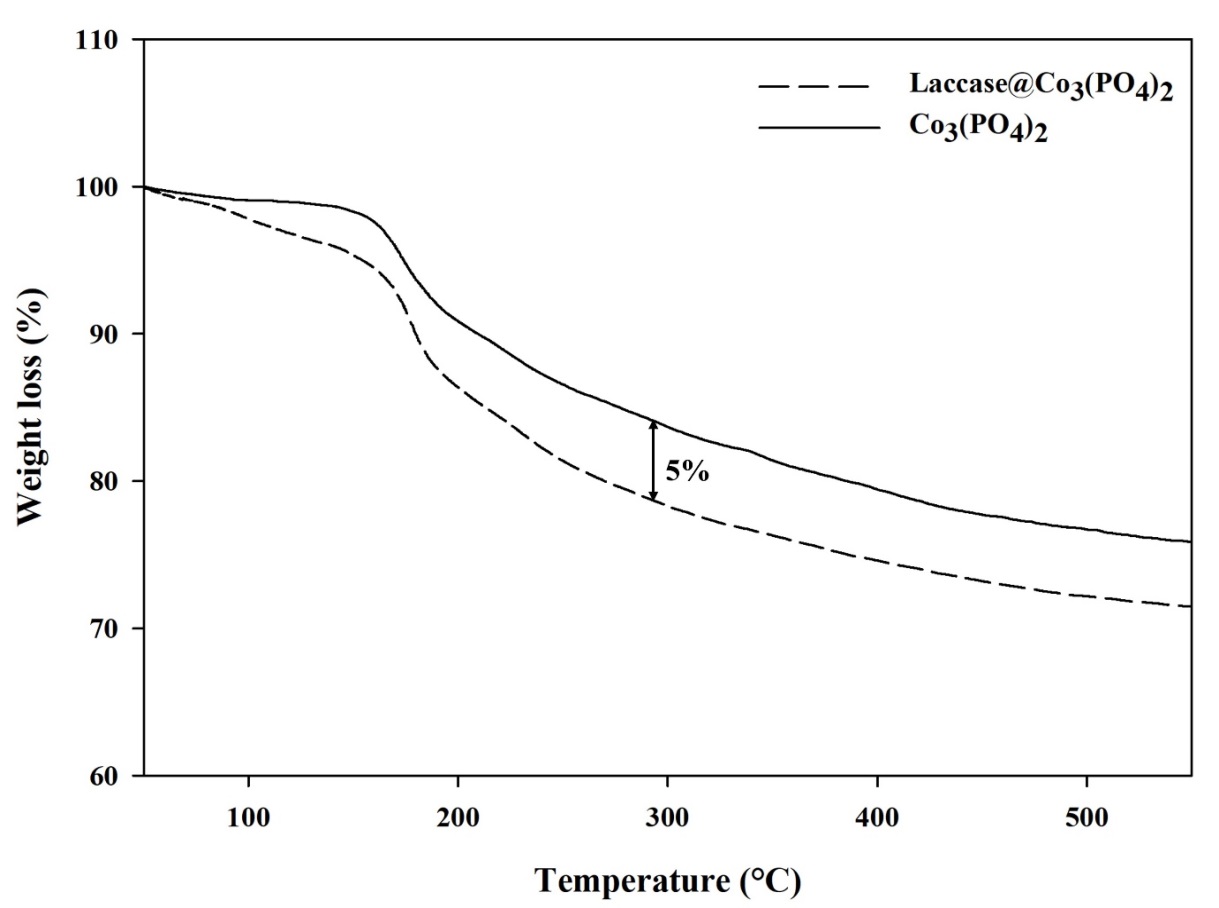


**Figure S8.** Thermogravimetric analysis of laccase@Co_3_(PO_4_)_2_•HNFs and Co_3_(PO_4_)_2_. Laccase composed 5% of the weight of the synthesized HNFs. The experiment was conducted after the complete degassing of samples under N_2_ atmosphere with a heating rate of 10 °C/min.


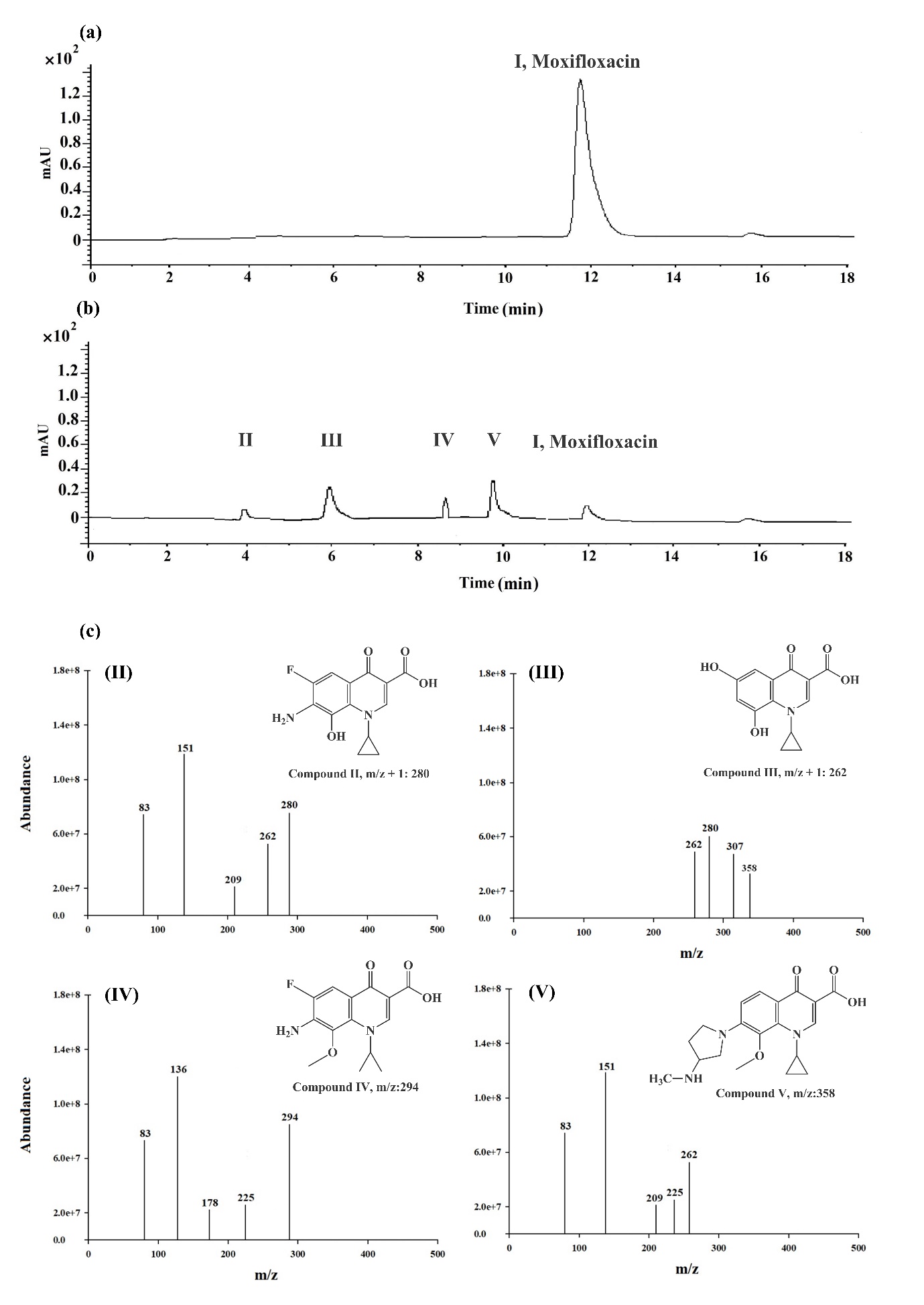


**Figure S9.** Liquid chromatography-mass spectrometry (LC-MS) analysis of moxifloxacin (R_t_ = 11.9 min) (a) and its corresponding detected degradation products (b). Mass spectra of the degradation products are presented as II) compound II (m/z: 280, R_t_ = 3.9 min), III) compound III (m/z: 262, R_t_ = 6 min), IV) compound IV (m/z: 294, R_t_ = 8.7 min), and V) compound V (m/z: 358, R_t_ = 9 min) (c).

**Table S1**. Weight percentage of elements in laccase@Co_3_(PO_4_)_2_•HNFs and Co_3_(PO_4_)_2_ measured by Energy dispersive X-Ray spectroscopy (EDX) analysis.

| **Type of material** | **Co (%)** | **O (%)** | **P (%)** | **N (%)** |
| --- | --- | --- | --- | --- |
| Laccase@Co_3_(PO_4_)_2_•HNFs | 42.3 | 29.5 | 16.7 | 11.5 |
| Co_3_(PO_4_)_2_•NFs | 54.7 | 10 | 31.3 | 4 |

**Table S2**. Mean pore diameter, total pore volume, and Brunauer-Emmett-Teller (BET) specific surface area of laccase@Co_3_(PO_4_)_2_•HNFs.

| **Material** | **Mean pore diameter (nm)** | **Total pore volume × 10^-3^ (cm^3^ g^–1^)** | **Specific surface area (m^2^ g^–1^)** |
| --- | --- | --- | --- |
| Laccase@Co_3_(PO_4_)_2_•HNFs | 29.5 | 128.5 | 17.4 |
| Co_3_(PO_4_)_2_•NFs | 22.3 | 98.1 | 17.6 |

**Table S3.** Growth inhibition percentage (GI%) of untreated and treated antibiotics with laccase@Co_3_(PO_4_)_2_ HNFs against four bacterial strains. Values are averages of three replicates ± standard deviation.

| **Antibiotics** | **Bacterial strains** | | | | | | | |
| --- | --- | --- | --- | --- | --- | --- | --- | --- |
|  | ***S*. *aureus*** | | ***S. epidermidis*** | | ***P*. *aeruginosa*** | | ***E*. *coli*** | |
|  | **Untreated** | **Treated** | **Untreated** | **Treated** | **Untreated** | **Treated** | **Untreated** | **Treated** |
| Moxifloxacin | 75.2 ± 0.6 % | 56.7 ± 1.2 % | 81.2 ± 0.4 % | 41.6 ± 0.7 % | 76.2 ± 0.3 % | 25.1 ± 2.1 % | 64.5 ± 0.4 % | 56.7 ± 1.2 % |

***** *p* < 0.05 vs. untreated
